# Generation and characterization of thiol-deficient *Mycobacterium tuberculosis* mutants

**DOI:** 10.1038/sdata.2018.184

**Published:** 2018-09-25

**Authors:** C. Sao Emani, M. J. Williams, P. D. Van Helden, M. J. C. Taylor, C. Carolis, I. J. Wiid, B. Baker

**Affiliations:** 1 NRF/DST Centre of Excellence for Biomedical Tuberculosis Research; South African Medical Research Council Centre for Tuberculosis Research; Division of Molecular Biology and Human Genetics, Faculty of Medicine and Health Sciences, Stellenbosch University, Tygerberg 8000, Cape Town, South Africa; 2 Barcelona Biomedical Research Park, Centre for Genomic Regulation, Biomolecular Screening & Protein Technologies Unit, 88 Dr.aiguider, 08003 Barcelona, Spain; 3 Central Analytical Facilities, Mass Spectrometry Unit, Stellenbosch University, Stellenbosch 7600, Cape Town, South Africa

**Keywords:** Bacterial transformation, Pathogens, Target identification, DNA recombination, DNA

## Abstract

Mycothiol (MSH) and ergothioneine (ERG) are thiols able to compensate for each other to protect mycobacteria against oxidative stress. Gamma-glutamylcysteine (GGC), another thiol and an intermediate in ERG biosynthesis has detoxification abilities. Five enzymes are involved in ERG biosynthesis, namely EgtA, EgtB, EgtC, EgtD and EgtE. The role of these enzymes in the production of ERG had been unclear. On the other hand, the enzyme MshA is known to be essential for MSH biosynthesis. In this manuscript, we describe the raw data of the generation and characterization of *Mycobacterium tuberculosis* (*M.tb)* mutants harbouring a deletion of the gene coding for each of these enzymes, and the raw data of the phenotypic characterization of the obtained thiol-deficient *M.tb* mutants. High throughput screening (HTS) of off-patent drugs and natural compounds revealed few compounds that displayed a higher activity against the thiol-deficient mutants relative to the wild-type strain. The mode of action of these drugs was further investigated. Raw data displaying these results are described here.

## Background & Summary

**Figure 1:**
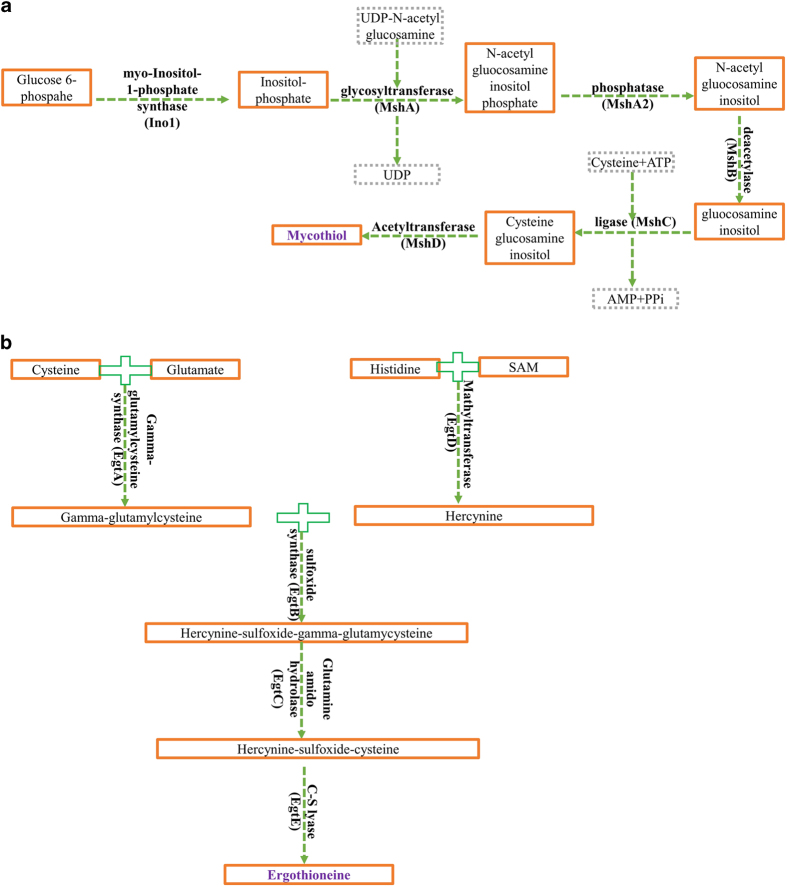
Simplified illustration of MSH biosynthesis and ERG biosynthesis. (**a**) The enzymes involved in MSH biosynthesis are Ino1, MshA, MshA2, MshB, MshC and MshD. MshA and MshC are essential for MSH biosynthesis. (**b**) The enzymes involved in ERG biosynthesis are EgtA, EgtB, EgtC, EgtD and EgtE^7^. EgtD and EgtB are essential for ERG biosynthesis. UPD: uridine diphosphate.

Mycothiol (MSH) and ergothioneine (ERG) are the major thiols of actinomycetes^1^. In order to better understand the role of these molecules, it is necessary to elucidate their biosynthesis, enzymes involved and the significance of each enzyme in the production of these thiols. Mycothiol biosynthesis has been described, requiring MshA, MshB, MshC and MshD, catalysing each a step (Fig. 1). However, only MshA and MshC are essential for MSH biosynthesis^2–4^. Mutants deficient in MshA and consequently MSH have been successfully generated in *Mycobacterium tuberculosis* (*M.tb*) (the causative agent of tuberculosis^5^). However, viable MshC-deficient *M.tb* mutants cannot be obtained, indicating, that MshC (but not MSH) is essential for the in vitro growth of *M.tb*^3,4,6^. On the other hand, ERG biosynthesis was speculative until recently when it was indicated that five enzymes EgtA, EgtB, EgtC, EgtD, EgtE catalyse each a step in ERG biosynthesis in *M. smegmatis*^7^ (Fig. 1). They are encoded by genes clustered in an operon (Table 1)^7^. Therefore, we performed a homology search to identify the corresponding genes in *M.tb* (Table 1).

This enabled us to generate *M.tb* mutants deficient in each enzyme, by generating an in frame deletion of each encoding gene^8^ (Table 1). In addition, in order to relate the role of ERG to MSH, a MSH-deficient mutant was generated through an in frame hygromycin marked deletion of *mshA*^8^ (Table 1). Intracellular^8^ and extracellular^9^ thiol levels of the generated mutants were measured by liquid chromatography tandem mass spectrometry (LC-MS).

**Figure 2:**
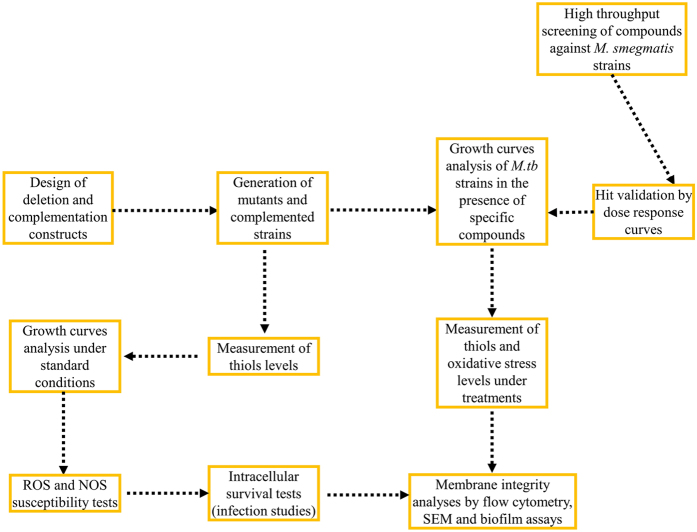
Workflow of experiments and data presented in this manuscript. ROS: reactive oxygen species, RNS: reactive nitrogen species.

The enzyme EgtA catalyses the formation of gamma-glutamylcysteine (GGC), an intermediate in ERG biosynthesis (Fig. 1)^7^ in mycobacteria and glutathione (GSH) biosynthesis in eukaryotes where it has anti-oxidative and anti-nitrosative detoxification roles^10,11^. The production of ERG was completely abolished in the ∆*egtD* and ∆*egtB* mutants, the production of GGC was completely abolished in the ∆*egtA* mutant which produced a very low level of ERG^8^, and the production of MSH was abolished in the ∆*mshA* mutant as previously shown^4,8^. Therefore, in order to explore the potential detoxification role of GGC in *M.tb*, we investigated the susceptibility of the generated GGC/ERG-deficient *ΔegtA* mutant relative to the MSH-deficient *ΔmshA* mutant and the ERG-deficient *ΔegtD* and *ΔegtB* mutants in various in vitro stress conditions^8^. Thiols biosynthetic enzymes are attractive drug targets. Nevertheless, standard tuberculosis (TB) regimens consist of more than one drug. However, the ∆*mshA* mutant is resistant to few current anti-TB drugs such as ethionamide and isoniazid^3,4,12^, suggesting that potential inhibitors of MshA such as UDP-(5F)-GlcNAc^13^ may not be suitable in combination with ethionamide, neither isoniazid. Therefore, in order to explore the possibility to repurpose off-patent drugs, and in order to explore the potential of natural compounds in a combination therapy with potential drugs that target thiols biosynthesis, we explore the activity of few compounds against the thiol-deficient mutants. An initial high throughput screening (HTS) of two libraries of compounds (off-patent drugs and natural compounds) against thiol-deficient *M. smegmatis* mutants was performed. *M. smegmatis* is a surrogate/model for *M.tb* during investigations^14,15^, because it is easier to manipulate since it is not pathogenic^14,15^. In addition, it facilitates experimental optimizations since it is fast-growing (doubling time of ~3-4 h relatively to a doubling time of ~24 h for *M.tb*)^14,15^, taking into account that the HTS required a series of optimizations. Few hits were obtained by the HTS^9^. Four hits were further expounded on *M.tb*, namely azaguanine (Aza), sulfaguanidine (Su), bacitracin (Ba) and fusaric acid (Fu). They were tested against thiol-deficient *M.tb* mutants by growth profiles analysis under the various treatments^9^. The growth inhibition of each compound on each strain was calculated from the growth curves raw data. To further understand the mechanism of action of these compounds in relation to thiols, the ability of these compounds to modulate the level of thiols (relative to the untreated controls) was investigated by LC-MS^9^. Since the general role of thiols is to protect cells against oxidative stress^16^, the ability of these compounds to generate oxidative stress (relative to the untreated controls) was also evaluated^9^. This was achieved by measuring the fluorescence intensity of the highly fluorescent 2',7'-dichlorofluorescein (DCF)^17^ that resulted from the cleavage by oxidation of the acetate groups of the non-fluorescent 2′,7′-dichlorofluorescein diacetate (DCFDA). Furthermore, para-aminobenzoic acid (pB) is able to antagonise sulphonamides, because they target the folate pathway where pB is an intermediate^18,19^. However, it was still unknown if it was able to antagonise Su and consequently implicating Su mode of action in the folate pathway. Therefore, the ability of pB to counteract the oxidative stress generated by Su was investigated and demonstrated^9^ with the aid of DCFDA. On the other hand, the ∆*egtD* mutant was resistant to Aza, a phenotype that was reversed in the complemented strain^9^, suggesting that EgtD is a possible target of Aza. In addition, Aza is able to inhibit the synthesis of S-adenosyl methioneine (SAM)^20^, which is the co-factor of EgtD (Fig. 1). However, it was impossible to rapidly investigate and clarify the mechanistic effect of the inhibition of the biosynthesis of SAM by Aza on the activity of EgtD. Since this happens intracellularly, it may implicate many other pathways. However, as a proof of concept, we investigated the activity of EgtD in the presence of a compound (chloroquine) that is known to bind to the SAM binding pocket of SAM-dependent enzymes^21^. As such, the ability of chloroquine to inhibit the activity of SAM-dependent enzymes such as EgtD and the human histamine methyltransferase (HNMT) was demonstrated^9^. Furthermore, to elucidate the source of extracellular thiols, and justify their extracellular detoxification role such as in the case of the detoxification of Ba, the membrane integrity of the thiol-deficient *M.tb* mutants was analysed^9^ by flow cytometry with the aid of propidium iodide (PI) that penetrates only damaged membrane and Syto 9 that penetrates any membrane^22^. Since Ba is a high molecular weight compound (impeding its penetration in mycobacteria)^23^, to further explain the susceptibility of the *ΔegtE* mutant to Ba, the alteration of the cell membrane lipid profile of this strain was investigated^9^. Mycobacteria with a thick lipid layer have a high tendency to aggregate in liquid cultures because of their hydrophobicity^24,25^. Therefore, we initially tested the ability of the Δ*egtE* mutant to form visual aggregates during the stationary phase of growth^9^. Osmium tetroxide is a stain used during microscopy, the higher the lipid content of the stained cells, the darker they appear (under the microscope)^26^. Therefore, we also tested the ability of this mutant to react with the osmium tetroxide stain using scanning electron microscopy (SEM)^9^. Finally we investigated its ability to form a biofilm^9^, since mycobacteria with altered lipid profiles hardly form biofilms^26,27^. Finally, we could relate the mode of action of these compounds to current anti-TB drug such as rifampicin and isoniazid^9^ through a checkerboard^28^ assay to determine their combined effect on mycobacterial growth. The workflow of the data presented in this study is displayed in Fig. 2. In these studies, we were able to determine the enzymes that were essential for ERG biosynthesis^8^. In addition, we were able to evaluate for the first time in *M.tb* the detoxification role of GGC as a potential thiol in relation to ERG and MSH^8^. Furthermore, we were able to give a brief evaluation of the physiology of the mutants deficient in each ERG biosynthetic enzyme and each thiol^8,9^. Finally, we were able to investigate the potential activity and mode of action of few compounds against the generated mutants in order to provide a rationale for further investigations into the possibility to repurpose/introduce these drugs in combination with potential inhibitors of thiol biosynthetic enzymes in a TB regimen. Raw data of these studies are described here and deposited in appropriate repositories (Data Citation 1–3).

## Methods

**Figure 3:**
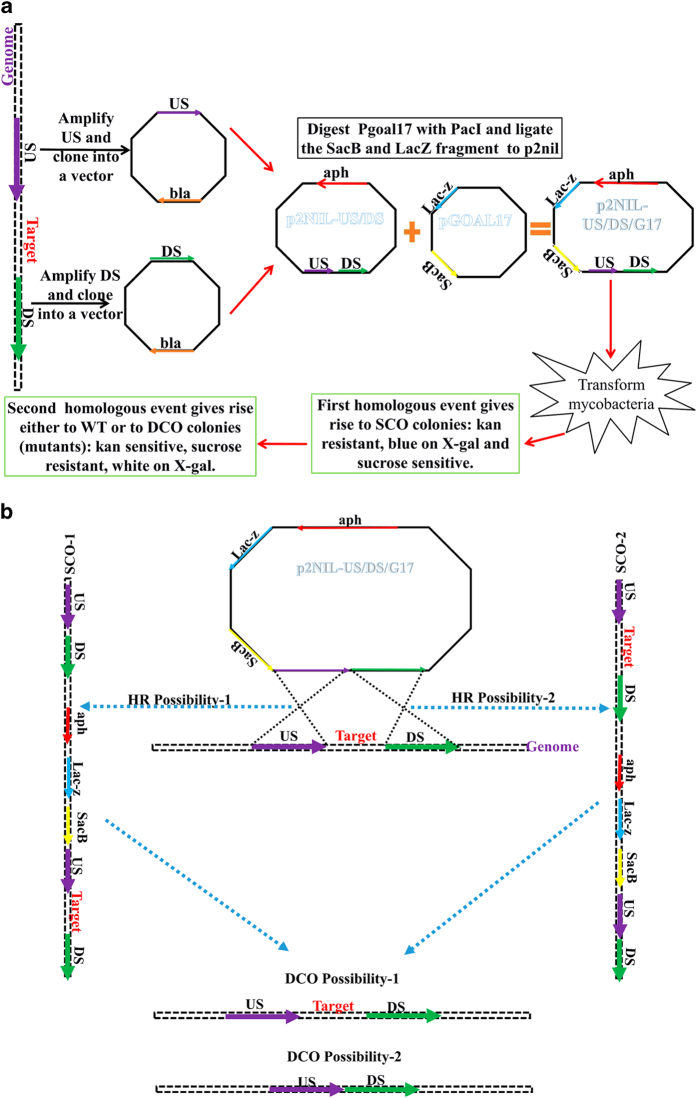
Gene deletion process adapted from previously described methods. (**a**) The upstream and the downstream regions of the targeted gene were amplified and subsequently cloned separately into any commercially available sub-cloning vector. Next, these fragments were excised by restriction enzyme digestions (RED) from these vectors and simultaneously ligated into a p2NIL vector that was previously linearized by RED. Then the PacI fragment of Pgoal17 (containing the sacB gene coding for levan sucrose for sucrose sensitivity and Lac-z gene coding for the β-galactosidase which breaks down X-gal to yield a blue product) was ligated to the p2NIL vector linearized by PacI. After confirmation of the integrity of the resulting construct by RED and by sequencing, it was used to achieve gene deletion as indicated. (**b**) After transformation of mycobacteria with the designed construct, a homologous recombination (HR) event occurs between the US (HR-1) or DS (HR-2) to give rise to single cross over (SCO) colonies that have incorporated the entire plasmid into their genome. The second HR event occurs after sub-culturing these colonies without antibiotics, giving rise to either a wild-type strain, or a strain with the deleted gene. Deletion was thereby confirmed by PCR and southern blotting analyses. Kan: kanamycin, X-gal: 5-bromo-4-chloro-3-indolyl-β-D-galactopyranoside, aph: kanamycin resistance gene, bla: ampicillin resistance gene; US: upstream, DS: downstream, WT: wild-type, SCO: single cross over, DCO: double cross over.

Generation of the *M.tb* mutants^8^ was achieved by a two-step homologous recombination (HR) method as previously described^29–31^ with few modifications (Fig. 3) (Data Citation 1). The following methods are expanded versions of descriptions in our related work^8^.

### Design of the deletion constructs

Primers^8^ used to amplify the upstream (US) and downstream (DS) regions of the target genes were designed with the programs known as DNAman (http://www.lynnon.com/) and GENtle (http://gentle.magnusmanske.de/). Designed primers fit the following criteria.

The upstream (US) and downstream (DS) regions were more or less 2,500 base pairs (bp) to minimize illegitimate HR (HR occurring between non-homologous regions).

Restriction sites (RS) were added at the extremities of each primer to enable subsequent cloning to the vector p2NIL. The chosen RS were found in the p2NIL multiple cloning site (MCS) but not in the US and DS regions that were amplified.

**Figure 4:**
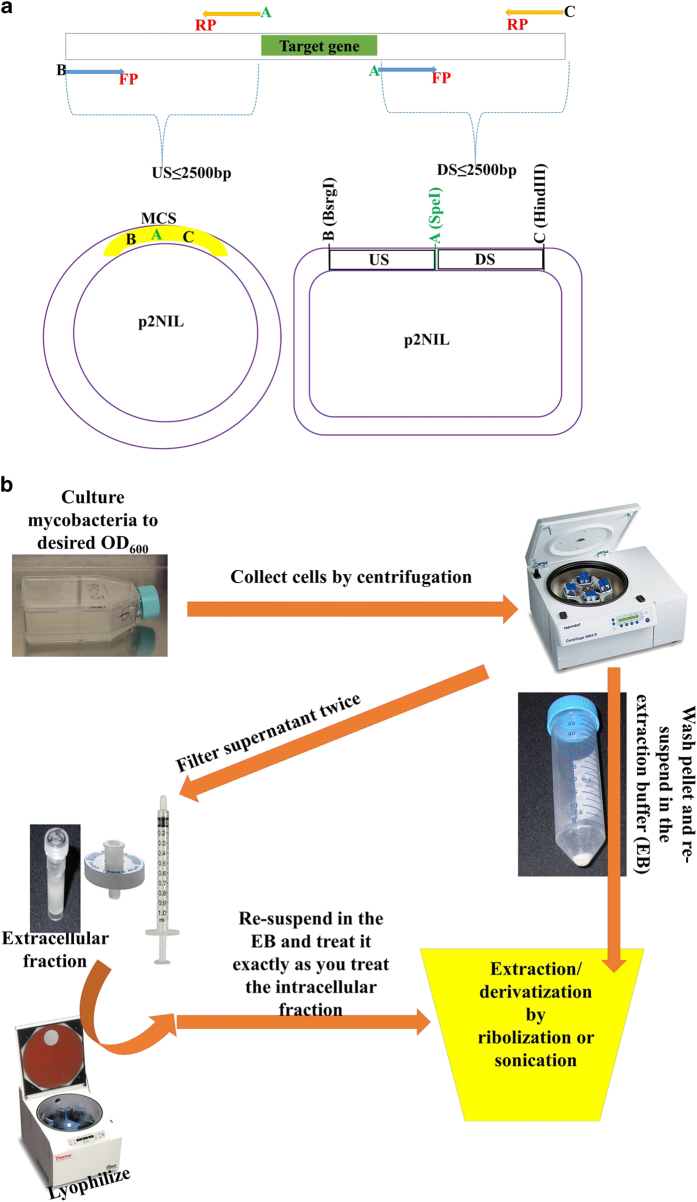
Experimental Designs. (**a**) Primers design for the deletion construct. The forward primer (FP) of the DS region carries RS-A (restriction site-A) identical to the RS of the reverse primer (RP) of the US region found in the p2NIL vector. RS-B and RS-C are found on either sides of RS-A. (**b**) Design of the metabolites extraction method. The supernatant of cultures after centrifugation is used for the quantitation of extracellular metabolites. The extract from the cells pellet is used for the quantitation of intracellular metabolites. Derivatization was performed either during or post extraction depending on the method.

The RS of the reverse primer of the US fragment was identical to the RS of the forward primer of the DS fragment to enable a two-way ligation of the two fragments into p2NIL (Fig. 4a).

The sequence of each primer designed to amplify the US and DS regions of each target gene has been described^8^. For the design of the specific constructs in this study, the restriction sites that met the criteria described above were for BsrgI (AauI) at the 5’-end of the forward primer (FP) of the US, SpeI at the 5’-end of the reverse primer (RP) of the US and the FP of the DS, HindIII at the 5’-end of the RP of the DS (Fig. 4a). These fragments were amplified by a High-Fidelity DNA Polymerase that enabled an accurate amplification of the targeted regions while taking into account the GC rich content of the *M.tb* genome.

Final constructs were screened by restriction enzyme digestions (RED) (Supplementary Fig. S1-S9) and by sequencing (Designed sequencing primers are P2nilF: GTTTACGAGAGAGATGATAG, P2nilR: ACGGTGCCTGACTGCGTTAG) (Data Citation 1). The plasmids with a satisfactory integrity that prevented *E.coli* from growing on sucrose containing plates were used as the allelic substrate to achieve deletion of the gene *egtA* (p2NILA/G17), *egtB* (p2NIlB/G17), *egtC* (p2NIlC/G17), *egtD* (p2NIlD/G17), *egtE* (p2NIlE/G17), *mshA* (p2NIlmshA/G17) (Data Citation 1). Deletion of *egtA*, *egtB*, *egtC, egtD*, *egtE* and *mshA* proceeded as described in Fig. 3. A colony or more of each mutant confirmed by PCR (Supplementary Fig. S10-S16) was further validated by Southern blotting^8^. Genomic extraction and southern transfer of digested genomic DNA was performed as previously described^32^, however, the detection steps were performed with the aid of the DIG High Prime DNA labelling and detection kit II and the DIG wash and block Buffer set (Roche Diagnostic GmbH Mannheim, Germany) according to manufacturer’s instructions. The methods used to design the complementation constructs have been described^8^ (Designed sequencing primers are pMV-R TGGCAGTCGATCGTACGCT, pMV-F: CCGGGCTGCAGGAATTCGATAT) (Data Citation 1).

### Sample preparation for the metabolomics analysis of the mutants

Sample preparation for the quantification of thiols by liquid chromatography tandem mass spectrometry (LC-MS) has been described in our previous work^8,9^ (Fig. 4b) and the raw mass spectra files are in the *Metabolights* repository (Data Citation 1). Derived and calculated data are described in the current work (Data Citation 1). Results derived from samples were prepared using two methods.

### Sample preparation method1

Briefly, exponential and stationary cultures were re-suspended in a pre-made extraction buffer ((40% acetonitrile (ACN) + 0.25 M perchloric acid (PA) + 2 mM ethylenediaminetetraacetic acid (EDTA)). After physical cell lysis by bead beating, the pH of the cell lysate was equilibrated to ~8 with ammonium bicarbonate (ABC). The suspension was further derivatized with a final concentration of 2 mM monobromobimane (mBBr, 60 °C for ~15 min). For the quantification of MSH using this method, sample were dithiothreitol (DTT)-treated prior derivatization (0.02 M DTT, 10 min at room temperature). For the quantification of ERG, the pH-equilibrated cell lysate was lyophilized and re-suspended in a resuspension buffer (2.48 mg/ml diethylenetriaminepentaacetic acid (DTPA) in 0.1% trifluoroacetic acid (TFA)). The suspension was added to a derivatization buffer (2.48 mg/ml DTPA + 48 mg/ml HEPES + 0.7 mM mBBr, pH 8.2) at a ratio of ~1:2 and incubated further for derivatization. For every extraction, the extracellular fractions were lyophilized and treated exactly as the intracellular fractions (Data Citation 1).

### Sample preparation method2

The first method consists of many steps that may have been the cause for the large variation between biological replicates, even when sample were DTT-treated as was the case of MSH quantification (Data Citation 1). However, the trend of difference between strains was conserved in most cases^8^. In addition, GGC, which was the most labile thiol, was hardly detected with method-1 (~1.4 Þg/10^10^ CFU in the wild-type versus ~14.6 Þg/10^10^ CFU in the ∆*egtB* mutant where it accumulates). This suggests that, the higher the number of steps during extraction, the more likely thiols are to oxidize and/or degrade. Therefore, we used a second relatively straightforward method to improve the quantification, focusing on the mutants that displayed the phenotype we were investigating^8^. In addition, we used the second method to quantify the level of thiols after various drugs treatment^9^ (Data Citation 1). The second method is described as follows. Briefly, as previously described with few modifications^4^, cell pellets and lyophilized extracellular fractions from stationary phase cultures were re-suspended in the lysis/derivatization buffer (50% warm ACN + 20 mM Hepes pH~8 + 2 mM mBBr) and sonicated in a water bath sonicator at 60 °C for ~30 min. This was further acidified with ~1 mM acetic acid and stored at −20 or −80 °C for the LC-MS analyses.

### Liquid chromatography tandem mass spectrometry for metabolomics analysis

Quantification by UPLC-ESI-MS/MS of MSH was performed with a Waters Acquity UPLC system coupled to a Waters Xevo TQ MS system (Waters Corporation, Milford, MA, USA). Compounds were separated on a Waters Acquity HSS T3 column (2.1 x 100mm; 1.7 μm particle diameter) at 40 °C using a gradient of 0.1% formic acid/water (solvent A) to 0.1% formic acid/acetonitrile (solvent B) at a flow rate of 0.35 ml/min. The acetonitrile concentration was increased linearly to 50% over 9 min, and thereafter to 100% after 10 min. The column was re-equilibrated for 3.5 min (the total run time was 14 min). The source capillary was held at 3 kV. The source and de-solvation temperatures were 140 °C and 400 °C, respectively. The de-solvation and cone gas flows were 600 and 50 liters/h, respectively. Quantification by UPLC-ESI-MS/MS of other metabolites (ERG, GGC and hercynine) was performed similarly with the same system. Compounds separation was performed using the same column specification at 40 °C using a gradient of 10 mM ammonium acetate/water (solvent A) to 49:49:2% acetonitrile:methanol:isopropanol (solvent B) at a flow rate of 0.34 ml/min. Solvent B was increased to 1% after 1 minute, followed by linear increases to 6% at 10.5 min, 15% at 12 min, 90% at 13 min where it was held until 13.5 min. The column was re-equilibrated for 3.4 min (the total run time was 17 min).

### Analysis of metabolomics raw data

For absolute quantifications such as that of ERG and GGC, the concentration derived from the standard curve was given in þg/μl. The total amount per vial was calculated by multiplying the given concentration by the total resuspension volume. This was further divided by the total volume of culture from which the sample was extracted, to have the final concentration per ml of culture, which was subsequently divided by the predetermined CFU per ml of culture for each strain (Data Citation 1).

For the relative quantifications such as that of MSH the given peak area was multiplied by half the total resuspension volume (assuming the peak area corresponds to 2 μl, since the injection volume was 2 μl) (Data Citation 1). However, for the quantification of hercynine, we considered an injection volume of 1** **μl (Data Citation 1). The total peak area was divided by the culture volume and subsequently by the CFU count as described above (Data Citation 1). Alternatively, ERG standard curve was used to deduce the absolute concentration of MSH in the data deriving from the second method^8,9^ (Data Citation 1).

### Flow cytometry analysis of mycobacteria membrane integrity

**Figure 5:**
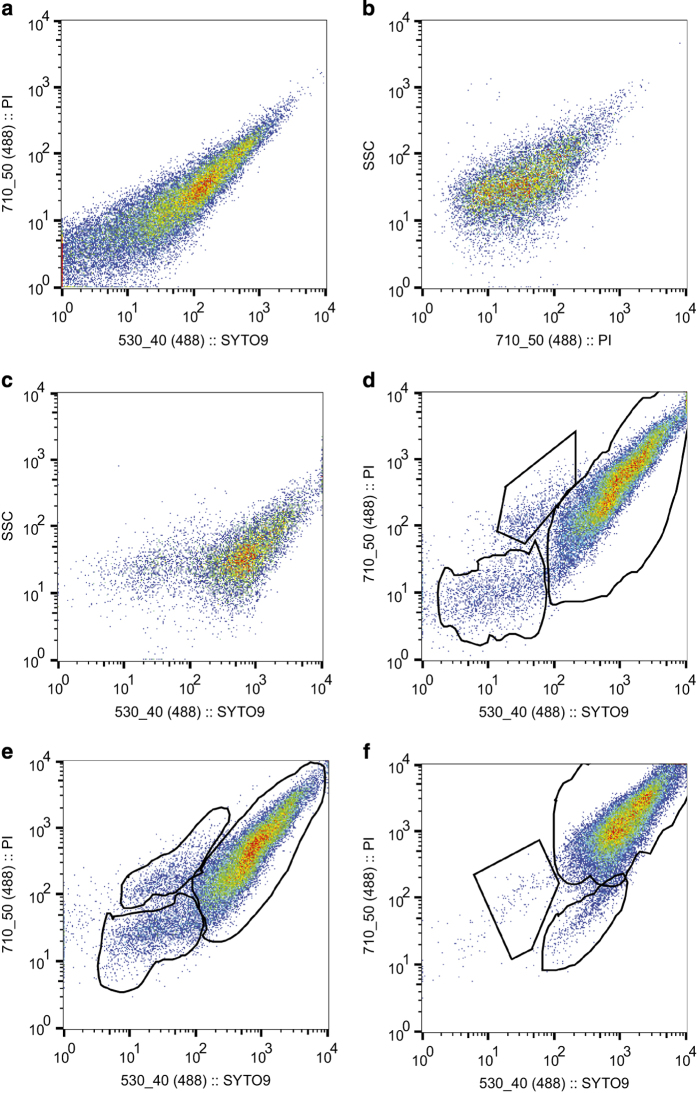
Flow cytometry analysis of *Mycobacterium tuberculosis.* (**a**) Syto9 versus PI plot of the unstained sample, indicating a low signal therefore minimal auto fluorescence or background fluorescence. (**b**) S*a*me sample stained only with PI. It can only penetrate cells with a damaged membrane either dead or not, which may explain the low signal since samples derived from actively growing cultures. (**c**) Sample stained with only Syto9. It penetrates every cell irrespective of their viability, which may explain the high signal. (**d**) Manual gating of population sub-sets using FlowJo of actively growing mycobacterial cultures stained with PI and Syto9. The population with a high Syto9 and PI signals was defined as the population of live cells with an intact membrane (LCIM). The population with a high PI signal and a relatively low Syto9 signal was defined as the population of dead cells (DC), while the population with a relatively low Syto9 and PI was considered as the population of live cells with a damaged membrane (LCDM). Every sample was analysed following that pattern. (**e**) Samples treated with 0.05% SDS for ~1 h displayed a slight increase of LCDM, indicating that a higher concentration of SDS or a longer exposure may damage a higher proportion of cell membrane. (**f**) When heat-treated, the DC population increased significantly.

The following method is an expanded version of description in our related work^9^. One millilitre of exponential *M.tb* cultures was pelleted, washed twice and re-suspended in 150 mM NaCl. One hundred microliters of the cell suspensions was added to BD Falcon tubes containing 977.4 μl of the running buffer (148 mM NaCl, 22 μM propidium iodide (PI) and 5 μM Syto9). Stained *M.tb* cells were analysed using a BD FACSJazz cell sorter (Becton Dickinson Biosciences, Belgium). Acquired data (Data Citation 3) were analysed using the program known as FlowJo (10.1r7). The instrument settings were adjusted according to the unstained sample (Fig. 5a) (Data Citation 3) that enabled to set the threshold and the single-stained samples (either with PI (Fig. 5b) or with Syto9 (Fig. 5c)) that enabled to set fluorescence compensations in order to correct for overlaps. Samples stained with both dyes were subsequently acquired after adjustments of the instrument settings (Fig. 5d). As controls, sodium dodecyl sulphate (SDS)-treated samples (~1h in 0.05% SDS) were included (Fig. 5e), based on previous studies indicating that SDS is able to damage bacterial membrane^33^. Heat-treated samples (80 °C for ~1h) (Fig. 5f) were added as additional controls based on previous indications that heat treatment under these conditions was detrimental to mycobacteria^34,35^. The wild-type and the mutants were subsequently analysed^9^ (Data Citation 1) (Data Citation 3).

### Growth curves generation

The following methods are expanded versions of descriptions in our related work^8,9^. Frozen stocks were used to start approximately 5 ml of culture in 25 cm^2^ vented tissue culture flasks (starter culture). After approximately a week or more of incubation at 37 ºC, the OD_600_ of the cultures was measured. This was used to calculate how much was needed to inoculate 40 ml of media (in a 75 cm^2^ vented tissue culture flask) to a starting OD_600_ of 0.05, supplemented with OADC (Oleic acid, albumin, dextrose and catalase) or ADS (albumin, dextrose and sodium chloride) with or without ERG^8^. The OD_600_ of each culture was recorded every second day (Data Citation 1). Alternatively, the starter culture was used to inoculate a smaller volume (10 ml) of culture media (in 25 cm^2^ vented tissue culture flasks) with or without the potential compounds identified through the high throughput screening^9^. The OD_600_ was recorded likewise every second day (Data Citation 1). *M. tuberculosis* strains were cultured without shaking at 37 °C; the flasks were placed and packed flat in sealable plastic containers that were double-sealed in plastic bags. The dose response curves of the *M. smegmatis* mutants were generated by a different method, with the aid of a plate reader in flat bottom dark 96-well plates as described^9^ (Data Citation 1).

### Susceptibility tests

Methods used to investigate mutants susceptibility to oxidative and nitrosative stress have been described^8^ (Data Citation 1). The method used to investigate their survival within THP1 macrophages has been described as well^8^ (Data Citation 1).

The investigation of the susceptibility of the mutants to antibiotics by growth curve analyses^9^ is described in the previous section. The growth inhibition was calculated by subtracting the OD_600_ of the treated culture from the OD_600_ of the untreated culture. Further dividing that by the OD_600_ of the untreated culture and finally multiplying by 100 ( Data Citation 1).

### Measurement of the levels of oxidative stress

This was achieved with the aid of the compound known as 2′,7′-dichlorofluorescein diacetate (DCFDA) as previously described^9,17^. An expanded version of our previous description^9^ is as follows. Briefly, DCFDA purchased from Sigma Aldrich (D6665-5G) was dissolved in DMSO to make a final concentration of 10 mM that was stored at -20 °C as the main stock. This was subsequently diluted to 1 mM stocks that were used to perform the analyses. Initial measurements consisted to stain 1 ml of mycobacteria cultures (grown to the stationary phase with or without treatment) with a final concentration of 10 μM of DCFDA. A volume of 100 μl was aliquoted in replicates in a dark 96-well plate (with an optical flat bottom). After incubation for 70 min at 37 ºC, measurements were performed at an excitation wavelength of 488 nm and emission wavelength of 520 nm with the aid of a plate reader (Data Citation 1). The relative fluorescence unit obtained was normalised with the CFU counts (Data Citation 1). Alternatively, in order to investigate the ability of pB to counteract the oxidative stress generated by Su, mycobacteria grown to an OD_600_~0.2 were treated with either Su, or pB or a combination of Su and pB. Since pB was dissolved in sterile double distilled H_2_O (2.5 mg/ml), H_2_0 was used to compensate for the volume of pB in the culture treated only with Su. On the other hand, since Su was dissolved 75% HCl (25 mg/ml), 75% HCl was used to compensate for the volume of Su in the culture treated only with pB. Treated cultures were immediately stained and the fluorescence intensity of DCF was measured every 10 min for 90 min at 37 °C (Data Citation 1).

### Investigation of the activity of EgtD and HNMT

Cloning, expression and purification of EgtD and HNMT have been described^9^. A more detailed description of the enzymatic assays from our previous work^9^ is as follows. The assays were performed with the aid of the SAMfluoro: SAM Methyltransferase Assay kit (G-Biosciences 9800 Page Avenue St. Louis, MO 63132-1429 USA) based on the following principle. During SAM mediated methylation by a methyltransferase, S-adenosylhomocysteine (AdoHcy) is released and converted to resofurin through a series of enzymatic reactions. The production of resofurin, which is proportional to the enzymatic activity, is therefore continuously quantified over time with a plate reader (the Infinite 200 PRO TECAN multimode microplate reader in this case) at an excitation wavelength of 530–540 nm and emission wavelength of 585–595 nm (Data Citation 1). Optimized specifications of the enzymatic reactions investigated in this study have been described^9^ (167 μM S-adenosylmethionine (SAM), 87 μM substrate and 42 nM enzyme). For cost effectiveness, initial investigations were performed to determine if the assays would function optimally at a scaled down reaction volume. This was achieved using 10 times less the manufacturer’s recommended volume (final reactions volume was 11.5 μl instead of 115μl performed in a dark flat bottom 384-well plate instead of a 96-well plate). The standard curves indicated a good correlation (See Supplementary Fig. S17), which prompted further investigations using smaller reaction volumes as described^9^.

### Drug combination studies (Checkerboard assays)

The results describing the effect of drugs in combination^9^ were obtained from drug combination studies performed as previously described^28^ but with few modifications as follows. The first compound was serially diluted from the 12^th^ to the 1^st^ row of a 96 well plate (first plate). Then the second compound was serially diluted from the H-column to the A-column of another 96 well plate in 7H9 supplemented with ADS (Second plate). Further, 50 μl of the diluted compound from every well of the first plate was transferred in the same order to every row except the 1^st^ row (where 50 μl of 7H9 was added instead, this served as the MIC control of the second compound) of a U-bottom 96 well plate (third plate). In addition, 50 μl of the serially diluted compound of the second plate was transferred to the third plate excluding the A-column (where 50 μl of 7H9 was added instead, this served as the MIC control of the first compound). Finally, 50 μl of early logarithmic phase mycobacterial cultures re-suspended to an OD~0.01 was added in every well of the third plate to make up a final volume of 150 μl. This was incubated for ≥7 days. Results were analysed by looking at the mycobacterial pellet formed at the bottom of the U-bottom plate or with the aid of the growth indicator resazurin^36^. For bactericidal compounds, the MIC (minimum inhibitory concentration) was determined as the concentration of the well with complete growth inhibition that was next to the well where growth was obvious. For bacteriostatic compounds, the MIC was determined as the concentration of the well with the smallest bacterial pellet size that was next to a bigger pellet size. The MIC of each compound (not in combination) previously determined by the broth micro dilution method^37^ was confirmed during the checkerboard assay from column-A (first compound) and the first row (second compound). Finally, the MIC in combination was determined from other wells.

### Aggregation test during the stationary phase

The *∆egtE* mutant and the wild-type CDC1551 strains were cultured to the stationary phase (for 4-8 weeks). Culture were transferred to 50 or 15 ml tubes and allowed to stand for at least five minutes in order to enable decantation of aggregates^9^ (Data Citation 1).

### Investigation of biofilm formation by *Mycobacterium tuberculosis*

The investigation of the ability of *M.tb* to form a biofilm^9^ was performed as previously described with slight modifications^26^. As we previously described^9^, it was perform as follows. Late log phase cultures were washed twice with an equal volume of Sauton’s medium. These cells were used to inoculate 50 ml of Sauton’s media to an OD_600_ of 0.01 in 250 ml sterile polypropylene bottles with tight caps (with no filters in order to prevent gas escape). Then it was incubated at 37 °C in a CO_2_ incubator for 3 to 6 weeks (Data Citation 1).

### Scanning electron microscopy

The investigation of the depletion of the lipid constituents of the membrane of the *∆egtE* mutant by scanning electron microscopy (SEM)^9^ was performed as follows. Late log phase liquid cultures were washed twice in an equal volume of Sauton’s medium and plated on 7H11 plates. The lawn of cells formed on the plate at ~3 weeks of incubation at 37 °C was scraped off, re-suspended in 3.7% formaldehyde and incubated at 4 °C overnight. The samples were cryofixed, immersed in a dry, pre-cooled (−85 °C) cocktail of 2% osmium tetroxide (OsO4) (dissolved in anhydrous acetone) kept in a Leica AFS Automatic Freeze-Substitution unit and subsequently analysed by SEM^38,39^.

## Data Records

The raw Flow Cytometry Standard files (FCS)^9^ are deposited in the *FlowRepository* (Data Citation 3). The metabolomics raw spectra files are deposited in the *Metabolights* repository (Data Citation 2). The following files and raw data are deposited in the *figshare* repository (Data Citation 1).

Raw and calculated data describing the metabolomics results

The empty cloning vectors, deletion and complementation constructs^8^ designed with the program GENtle.

The sequencing results of the designed constructs^8^ from different set of primers (taking into account that the first 50–100 nucleotides from the sequencing results are usually spurious).

Raw data of every growth curve presented in these studies^8,9^ (standard conditions (OADC or ADS supplement) and under treatments).

CFU counts raw data and calculated survival percentage of the various susceptibility tests performed in these studies^8,9^.

Raw data of the activity assays of EgtD and HNMT^9^

Aggregation, biofilm, and SEM pictures from various biological replicates of tested *M.tb* strains^9^.

## Technical Validation

Constructs generated in this study are sequenced (Data Citation 1). They were also screened by RED (see Supplementary Fig. S1-S9).

Mutants were screened by PCR (see Supplementary Fig. S10-S16) and confirmed by southern blotting^8^, sequence of primers and probes used for screenings have been described^8^.

Metabolomics analyses were performed from samples deriving from two different cultures of each strain (cultures started and cells harvested on different days) (Data Citation 1). Results were further validated in specific strains using another extraction-derivatization method from another set of two different cultures^8^ (Data Citation 1). Intracellular and extracellular fractions were obtained from the same biological samples and were derivatized in the same buffer, to allow a rational comparison of the data obtained from both fractions. In order to ensure a rational comparison of thiols levels between different *M.tb* strains, their samples were extracted the same day with the same buffer, and analysed during the same LC-MS run, to rule out any difference that may have arisen because of the difference in the LC-MS or sample preparation conditions^8,9^. To minimize spurious interpretations of the levels of thiols under treatment^9^ (that may have arisen from dead mycobacteria), treatment was introduced at the start of the culture (OD_600_ ~0.05) and the levels of thiols were measured after ~ two weeks of mycobacterial growth under the selective pressures. This was in order to determine the regulation of the thiols production when the mycobacteria are cultured in the presence of the compounds relative to the untreated controls (Data Citation 1). That’s why it was not investigated at the compounds MICs but rather at their average MIC, since this was the average of the minimum concentration that completely inhibited growth and the maximum concentration that did not inhibit growth^9^ determined by the broth micro dilution method^37^. For more accuracy, the levels of thiols in these studies^8,9^ were normalized with the CFU counts in order to directly relate their levels to the viable mycobacterial cells (Data Citation 1).

Likewise, the fluorescence intensity of DCF measured in treated cultures was normalised with the CFU counts of each culture^9^ (Data Citation 1). In addition, the level of ROS and thiols were measured from the same biological samples in order to be able to directly relate the level of thiols to the level of ROS measured in each treated sample^9^ (Data Citation 1). However, during the investigation of the counteracting oxidative stress effect of pB on Su^9^, it was not necessary to normalize the fluorescence intensity of DCF with the CFU counts. Since the different conditions were investigated in equal volumes of the same sample (derived from the same culture, *M.tb* wild-type at OD~0.2) and measurements were performed immediately after adding the DCFDA. Thus, the counteracting effect of Su and pB was monitored over time (Data Citation 1). Nevertheless, we ensured that a uniform percentage of HCl was included in every tested condition (since Su was dissolved in HCl) in order to rule out any observation that may have been associated with HCl. In addition, it was found that HCl quenches the fluorescence of DCF, which further justified the addition of the same percentage of HCl in the controls.

Growth curve analyses and susceptibility tests were performed two to four times to ensure reproducibility of results^8,9^ (Data Citation 1).

To ensure the appropriate population gating^9^, heat-treated, SDS-treated, unstained untreated and finally single-stained (with either PI or Syto9) samples were included during flow cytometry analyses (Data Citation 3).

To ensure that the absence of aggregation of the *ΔegtE* CDC1551 mutant^9^ was not due to a high concentration of Tween 80 in the media, every tested strain was grown in the same media. The inability of the *∆egtE* mutant to aggregate was investigated more than once (Data Citation 1). Likewise, the inability of the *∆egtE* mutant to form a biofilm was investigated more than once, from different bacterial stocks cultured under different conditions (Data Citation 1).

To ensure that the dark colour intensity of the mycobacterial cells^9^ visualised by SEM was not different due to the instrument settings during visualization, they were visualised under the same specifications (Contrast: 53.3%, Brightness: 49.9%,) (Data Citation 1).

Untreated controls were added during all susceptibility tests that involved prolonged exposures (more than 48 h for *M.tb* or more than more than 3 h for *M. smegmatis*) to make sure that the observed differences were not due to growth differences (Data Citation 1).

Finally, the susceptibility of the *M.tb* or *M. smegmatis* strains to various compounds and the level of ROS were determined in cultures supplemented with either ADS (for *M.tb* strains) or GS (0.85% NaCl, 0.2% glucose) (for *M. smegmatis* strains)^9^. It was in order to avoid catalase (that is able to react with ROS, acting as an anti-oxidant^40^) in the OADC supplement that could compromise the integrity of the results of the specific experiments that relied on the ability of the mycobacteria to counteract ROS and the ability of the tested compounds to generate ROS.

## Usage Notes

Researchers interested in reproducing the results generated in these manuscripts. Researchers interested in reproducing/performing similar experiments in other contexts. Raw data files are either in excel or Graph Pad Prism files. Sequencing results can be analysed with any gene manipulation software and constructs maps can be open with the program GENtle or any other gene manipulation software.

The library of compounds used for the high throughput screening are the PRESTWICK chemical (1,280 compounds) and the PRESTWICK phytochemical (320 compounds) libraries. The chemical library consists of chemical and pharmacological diverse off-patent drugs with known bioavailability and biosafety data in humans. The phytochemical library consists of natural compounds that mostly derived from plants.

## Additional information

**How to cite this article**: Emani, C. S. *et al*. Generation and characterization of thiol-deficient Mycobacterium tuberculosis mutants. *Sci. Data* 5:180184 doi: 10.1038/sdata.2018.184 (2018).

**Publisher’s note**: Springer Nature remains neutral with regard to jurisdictional claims in published maps and institutional affiliations.

## Supplementary Material



Supplementary Information

## Figures and Tables

**Table 1:** Gene annotations and mutants used in this study.

**Enzyme**	**EgtA**	**EgtB**	**EgtC**	**EgtD**	**EgtE**	**MshA**
	**Gamma-glutamylcysteine synthase**	**sulfoxide synthase**	**glutamine amidohydrolase**	**S-adenosylmethionine-dependent methyltransefrase**	**C-S lyase**	**Mycothiol glycosyl transferase**
Gene	*egtA*	*egtB*	*egtC*	*egtD*	*egtE*	*mshA*
ORF^a^ in *M. smegmatis*	*MSMEG_6250*	*MSMEG_6249*	*MSMEG_6248*	*MSMEG_6247*	*MSMEG_6246*	*MSMEG_0924*
ORF in *M.tb* (H37Rv)	*Rv3704c*	*Rv3703c*	*Rv3702c*	*Rv3701c*	*Rv3700c*	*Rv0486*
ORF in *M.tb* (CDC1551)	*MT3807*	*MT3806*	*MT3805*	*MT3804*	*MT3803*	*MT0504*
*M. smegmatis* mutants	_	_	_	Unmarked HR^b^ mutant, ∆*egtD*^12^	_	Kanamycin marked, transposon mutant ∆*mshA*^2^
*M.tb* mutants (H37Rv)	_	Unmarked HR mutant, *∆egtB*^8^	_	_	Unmarked HR mutant, ∆*egtE*^8^	
*M.tb* mutants (CDC1551)	Unmarked HR mutant ∆*egtA*^8^	Unmarked HR mutant ∆*egtB*^8^	Unmarked HR mutant ∆*egtC*^8^	Unmarked HR mutant ∆*egtD*^8^	Unmarked HR mutant ∆*egtE*^8^	Hygromycin marked HR mutant, ∆*mshA*^8^

^a^Open reading frame.

^b^Homologous recombination.
