# Supplementary material for: Generation and characterization of thiol-deficient Mycobacterium tuberculosis mutants
Source: Sci Data. 2018 Sep 25;5:180184. doi: 10.1038/sdata.2018.184 (PMC6154287; doi:10.1038/sdata.2018.184)
Supplement: Supplementary Information [file sdata2018184-s2.docx]

**Generation and characterization of thiol-deficient *Mycobacterium tuberculosis* mutants**

C. Sao Emani^a^*, MJ. Williams^a^, PD.Van Helden^a^, MJC. Taylor^b^, C. Carolis^c^, IJ. Wiid^a^, B. Baker^a^*

^a^DST-NRF Centre of Excellence in Biomedical Tuberculosis Research; SAMRC Centre for Tuberculosis Research; Division of Molecular Biology and Human Genetics; Department of Biomedical Sciences; Faculty of Medicine and Health Sciences; Stellenbosch University; Cape Town, South Africa

^b^Central Analytical Facilities, Mass Spectrometry Unit, Stellenbosch University; Cape Town, South Africa

^c^Barcelona Biomedical Research Park, Centre for Genomic Regulation, Biomolecular Screening & Protein Technologies Unit, 88 Dr.aiguider, 08003 Barcelona, Spain

*karallia@sun.ac.za, brubaker@sun.ac.za

**2** | Page

Contents

[Supplementary Figure. S1 3](#_Toc516485838)

[Supplementary Figure. S2 4](#_Toc516485839)

[Supplementary Figure. S3 5](#_Toc516485840)

[Supplementary Figure. S4 6](#_Toc516485841)

[Supplementary Figure. S5 7](#_Toc516485842)

[Supplementary Figure. S6 8](#_Toc516485843)

[Supplementary Figure. S7 9](#_Toc516485844)

[Supplementary Figure. S8 10](#_Toc516485845)

[Supplementary Figure. S9 11](#_Toc516485846)

[Supplementary Figure. S10 12](#_Toc516485847)

[Supplementary Figure. S11 13](#_Toc516485848)

[Supplementary Figure. S12 14](#_Toc516485849)

[Supplementary Figure. S13 15](#_Toc516485850)

[Supplementary Figure. S14 16](#_Toc516485851)

[Supplementary Figure. S15 17](#_Toc516485852)

[Supplementary Figure. S16 18](#_Toc516485853)

[Supplementary Figure. S17 19](#_Toc516485854)

# Supplementary Figure. S1


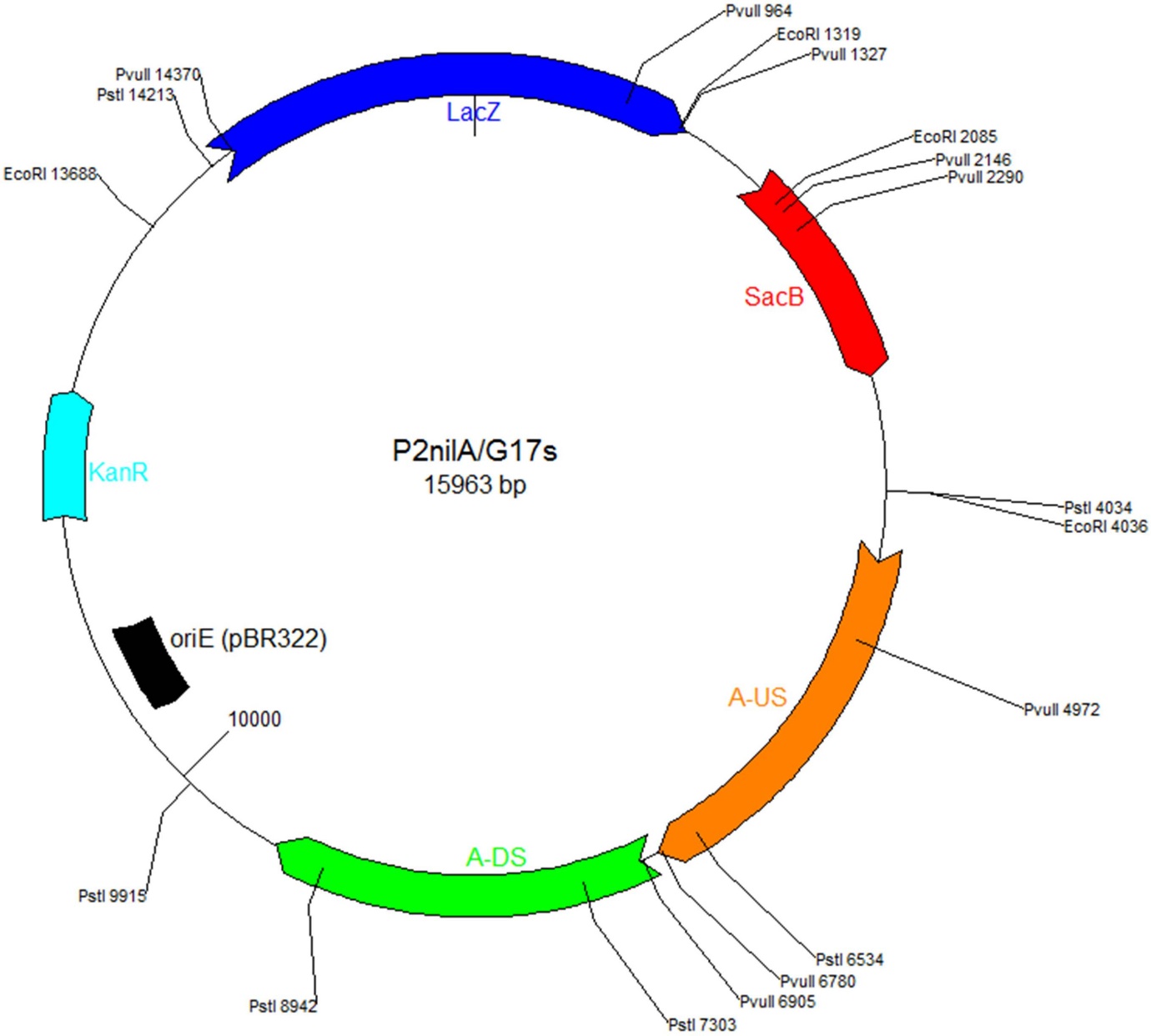


**Figure S1. Virtual design of *egtA* deletion construct (p2nilA/G17).** Enzymes that cut within the inserts (A-US, A-DS) and out of the inserts were chosen to investigate the construct integrity by restriction enzyme digestions (RED) (PVuII, PstI and EcoRI) (Data Citation 1)

# Supplementary Figure. S2


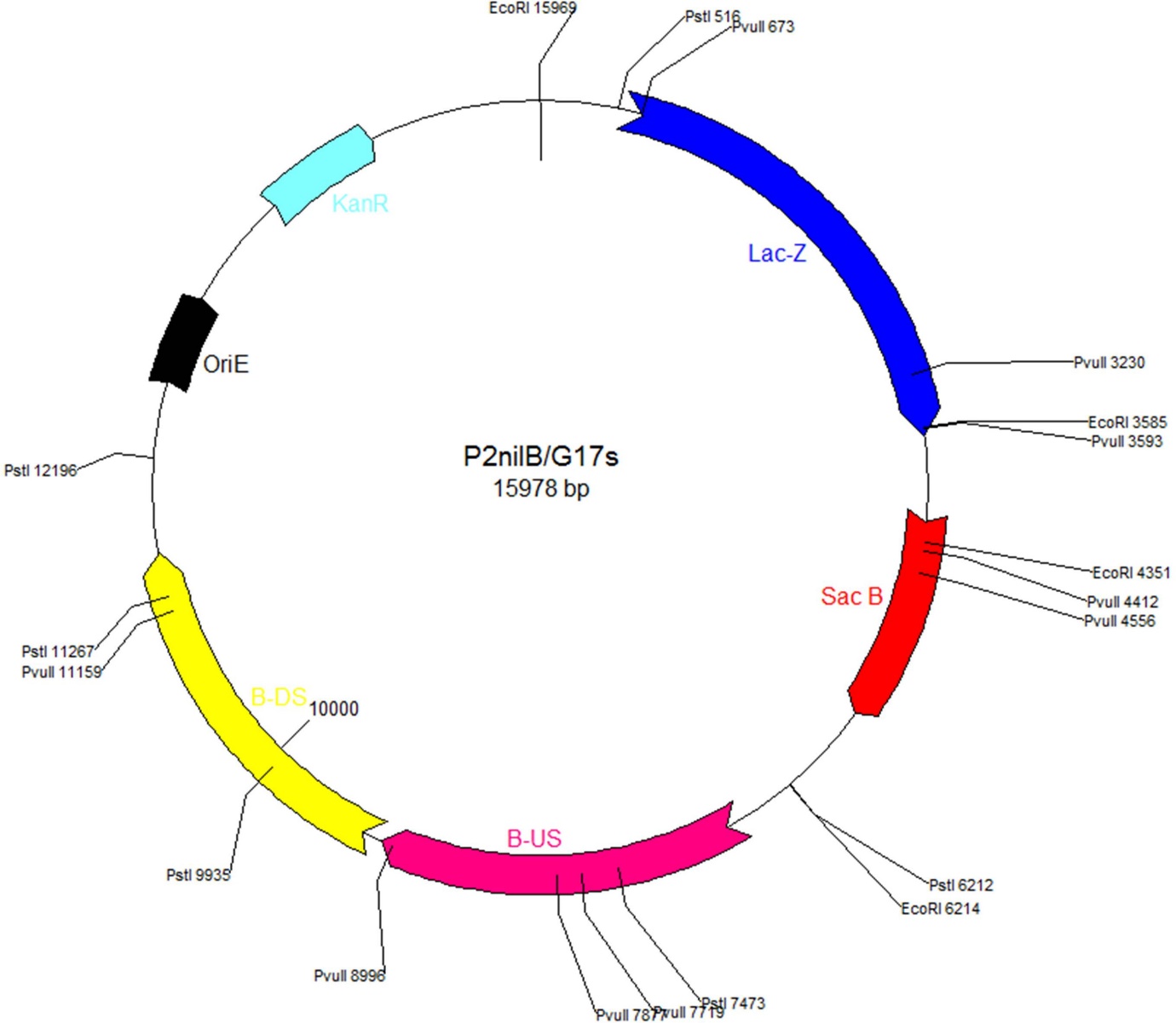


**Figure S2. Virtual design of *egtB* deletion construct (p2nilB/G17).** Enzymes that cut within the inserts (B-US, B-DS) and out of the inserts were chosen to investigate the construct integrity by RED (PVuII, PstI and EcoRI) (Data Citation 1).

# Supplementary Figure. S3


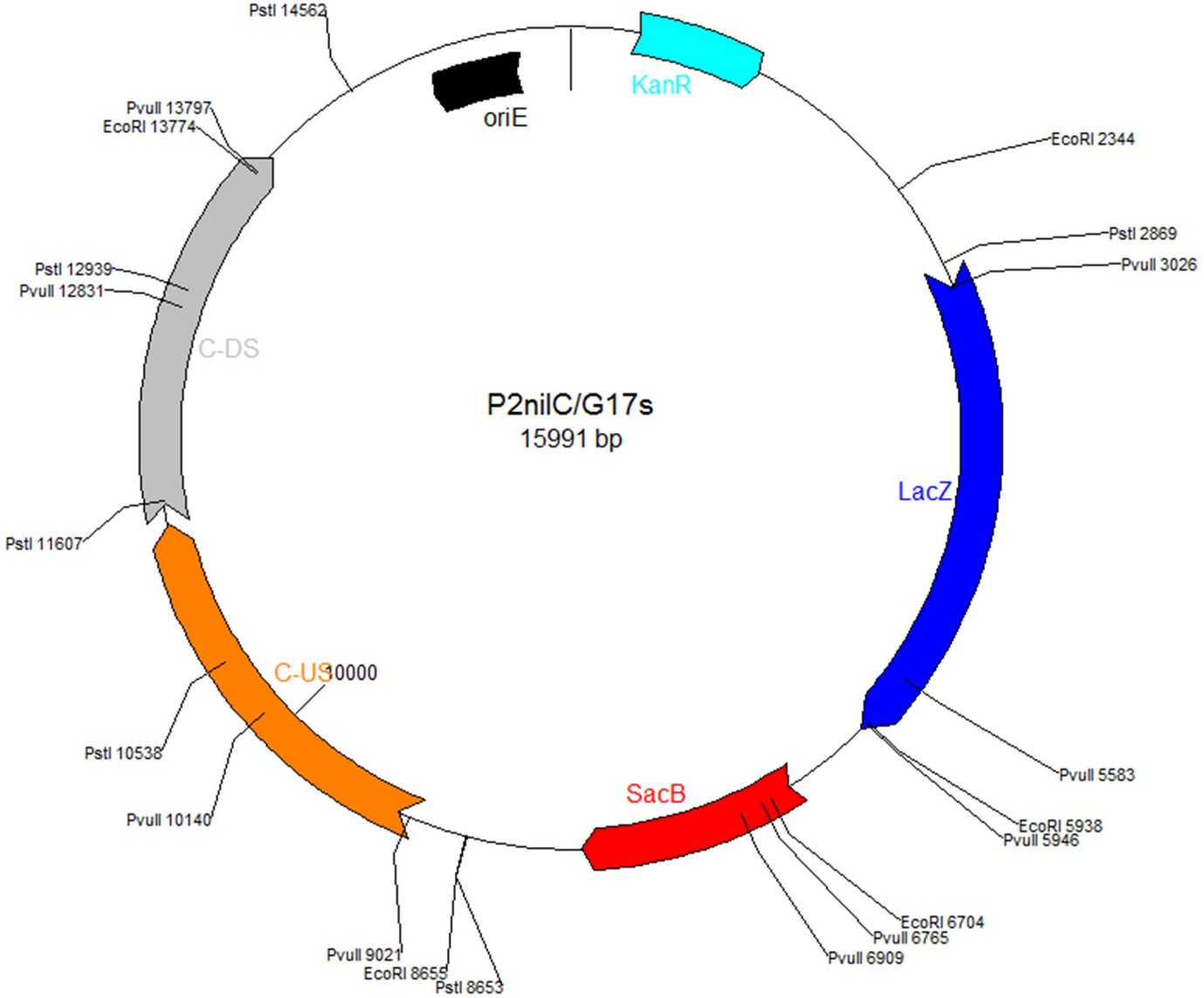


**Figure S3. Virtual design of *egtC* deletion construct (p2nilC/G17).** Enzymes that cut within the inserts (C-US, C-DS) and out of the inserts were chosen to investigate the construct integrity by RED (PVuII, PstI and EcoRI) (Data Citation 1).

# Supplementary Figure. S4


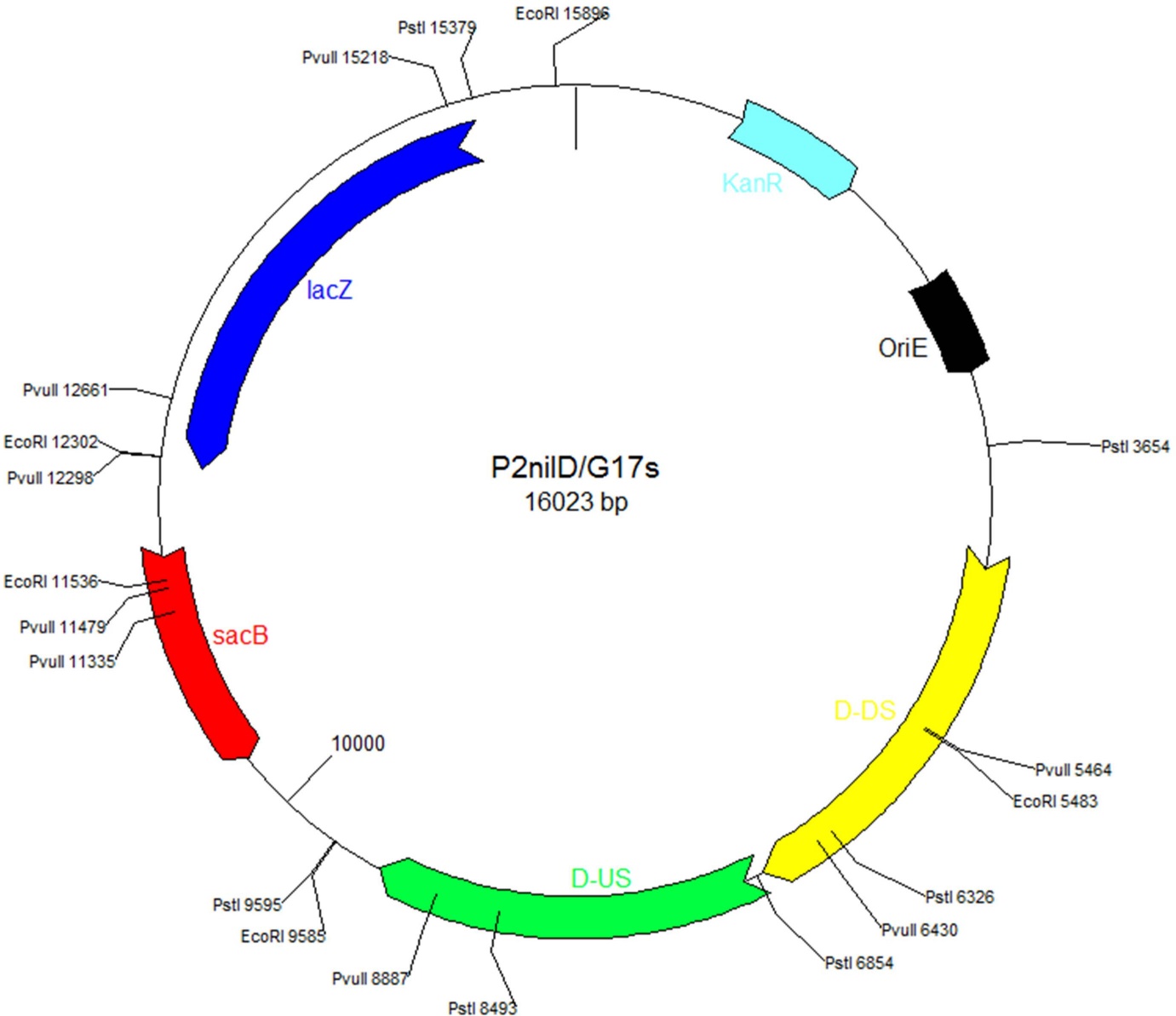


**Figure S4. Virtual design of *egtD* deletion construct (p2nilD/G17).** Enzymes that cut within the inserts (D-US, D-DS) and out of the inserts were chosen to investigate the construct integrity by RED (PVuII, PstI and EcoRI) (Data Citation 1).

# Supplementary Figure. S5


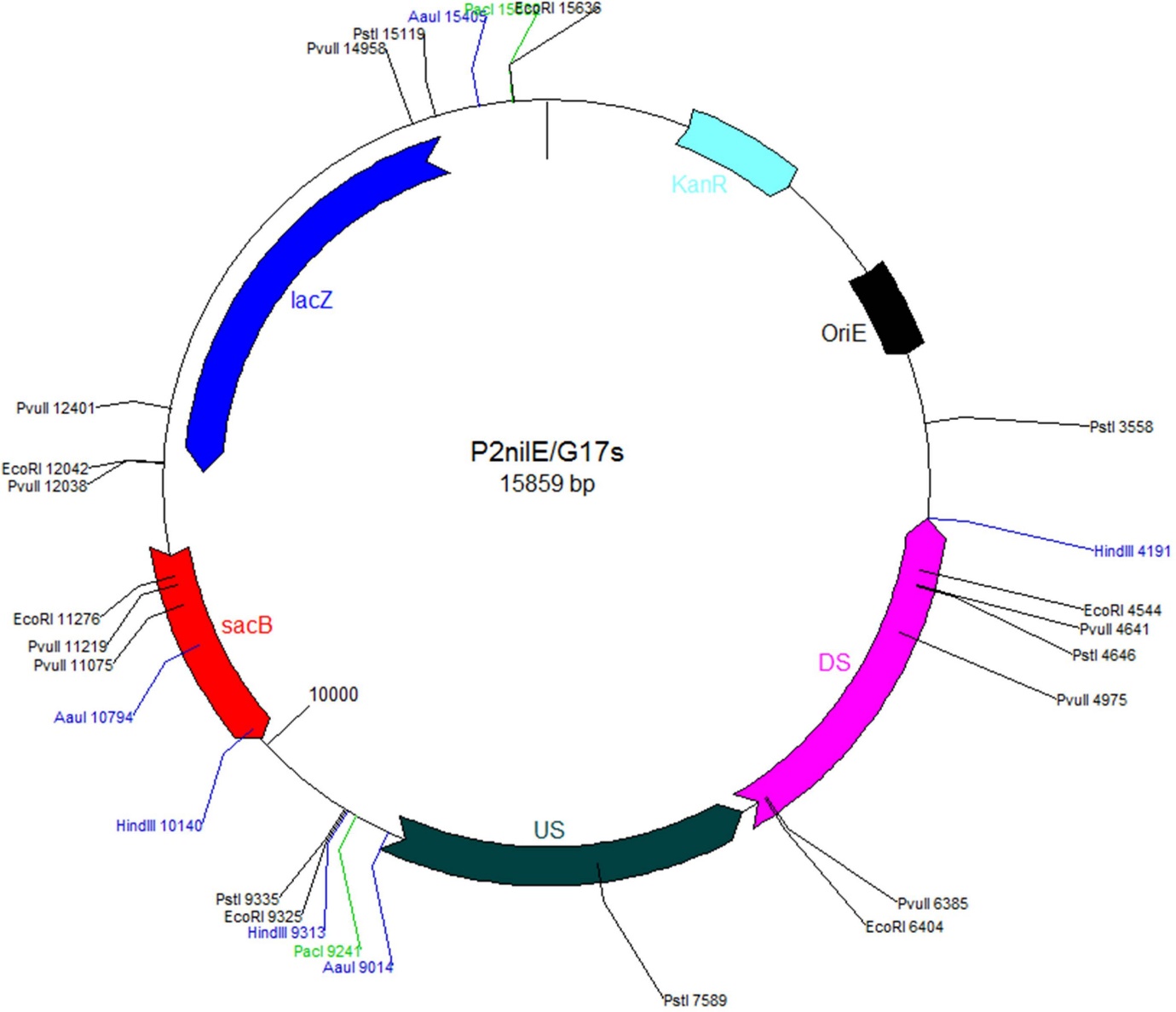


**Figure S5. Virtual design of *egtE* deletion construct (p2nilE/G17).** Enzymes that cut within the inserts (E-US, E-DS) and out of the inserts were chosen to investigate the construct integrity by RED (PVuII, PstI and EcoRI) (Data Citation 1).

# Supplementary Figure. S6


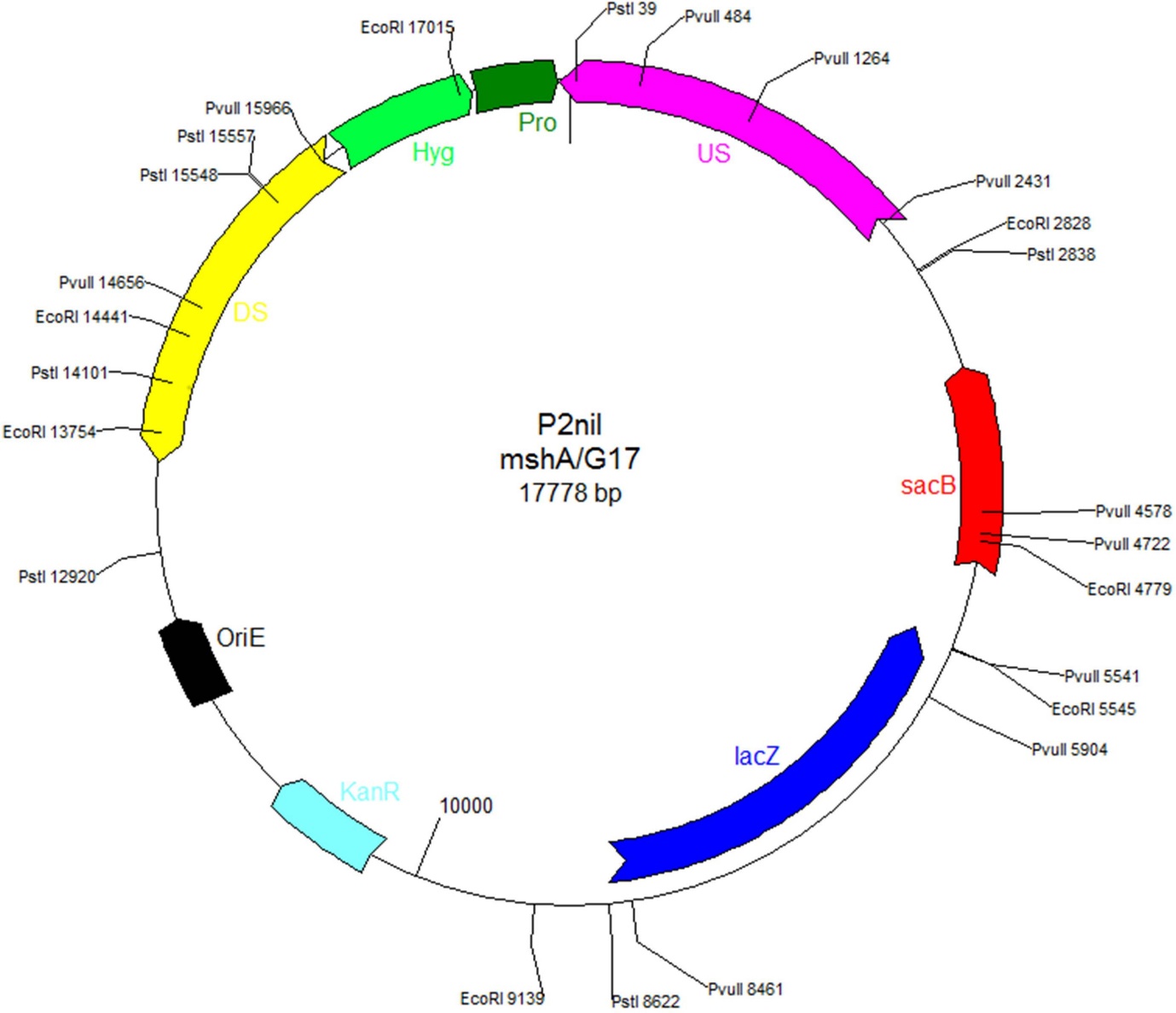


**Figure S6. Virtual design of *mshA* deletion construct (p2nilmshA/G17).** Enzymes that cut within the inserts (US, DS, Hyg, pro (promoter) ) and out of the inserts were chosen to investigate the construct integrity by RED (PVuII, PstI and EcoRI) ( Data Citation 1).

# Supplementary Figure. S7


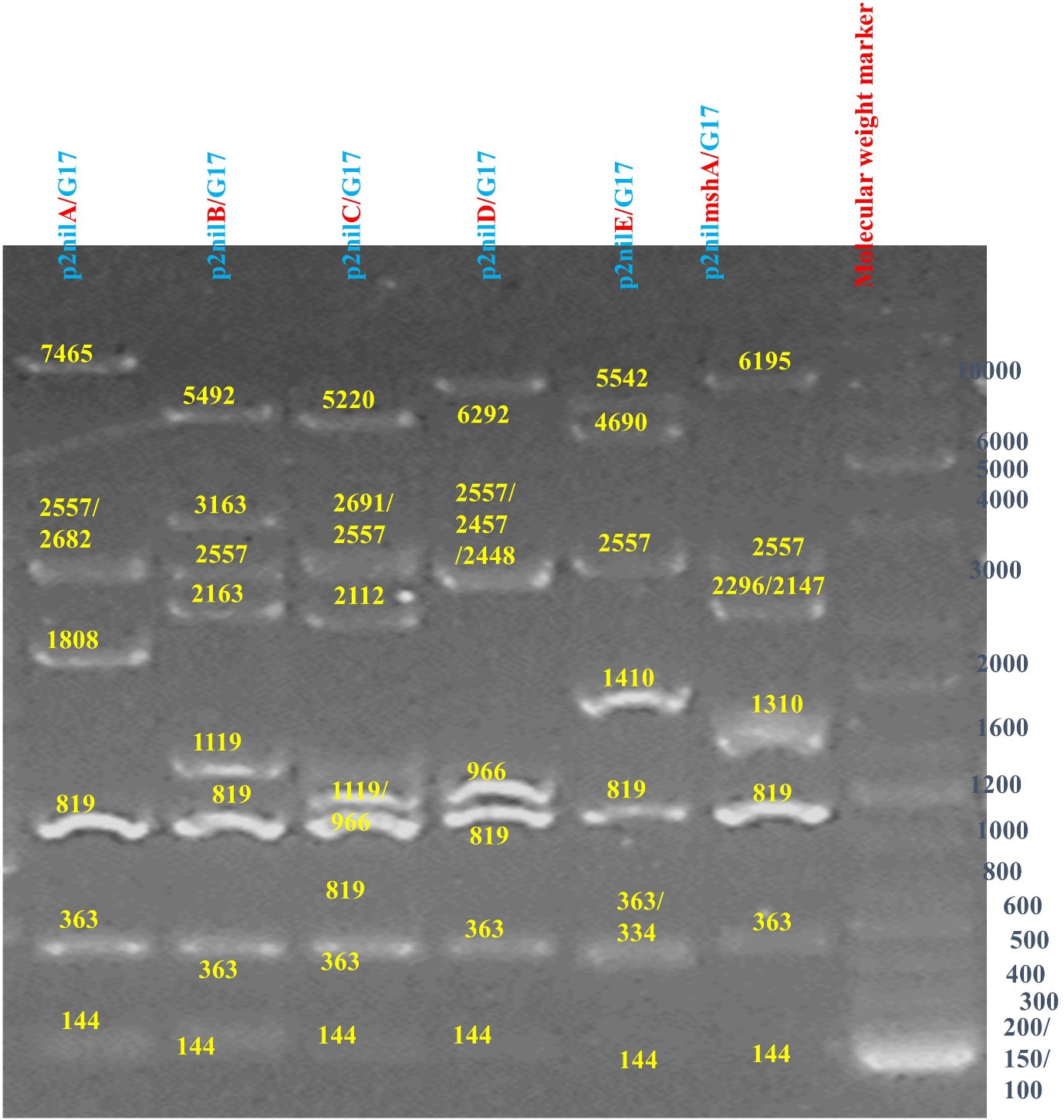


**Figure S7. Restriction enzyme digestion by PvuII of the generated** **constructs**. Expected bands according to the map in the program GENTle were obtained (Data Citation 1). Digestion of deletion constructs of the *egtA* first lane, *egtB* second lane, *egtC* third lane, *egtD* fourth lane, *egtE* fifth lane and *mshA* sixth lane, and molecular weight markers on the last lane.

# Supplementary Figure. S8


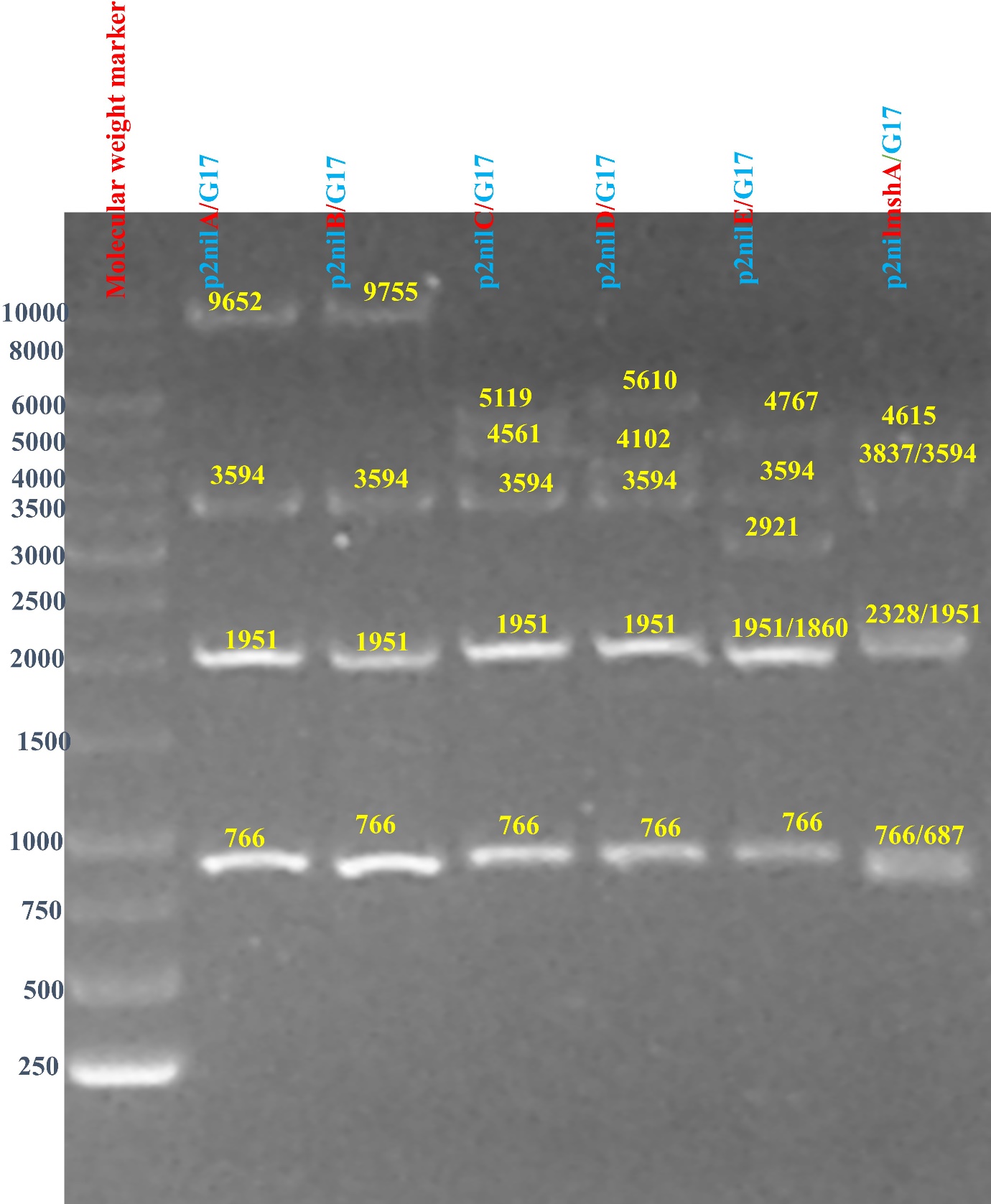


**Figure S8. Restriction enzyme digestion by EcoR I of the generated** **constructs**. Expected bands according to the map in the program GENTle were obtained (Data Citation 1). Digestion of deletion constructs of the *egtA* second lane, *egtB* third lane, *egtC* fourth lane, *egtD* fifth lane, *egtE* sixth lane and *mshA* last lane, and molecular weight markers on the first lane.

# Supplementary Figure. S9


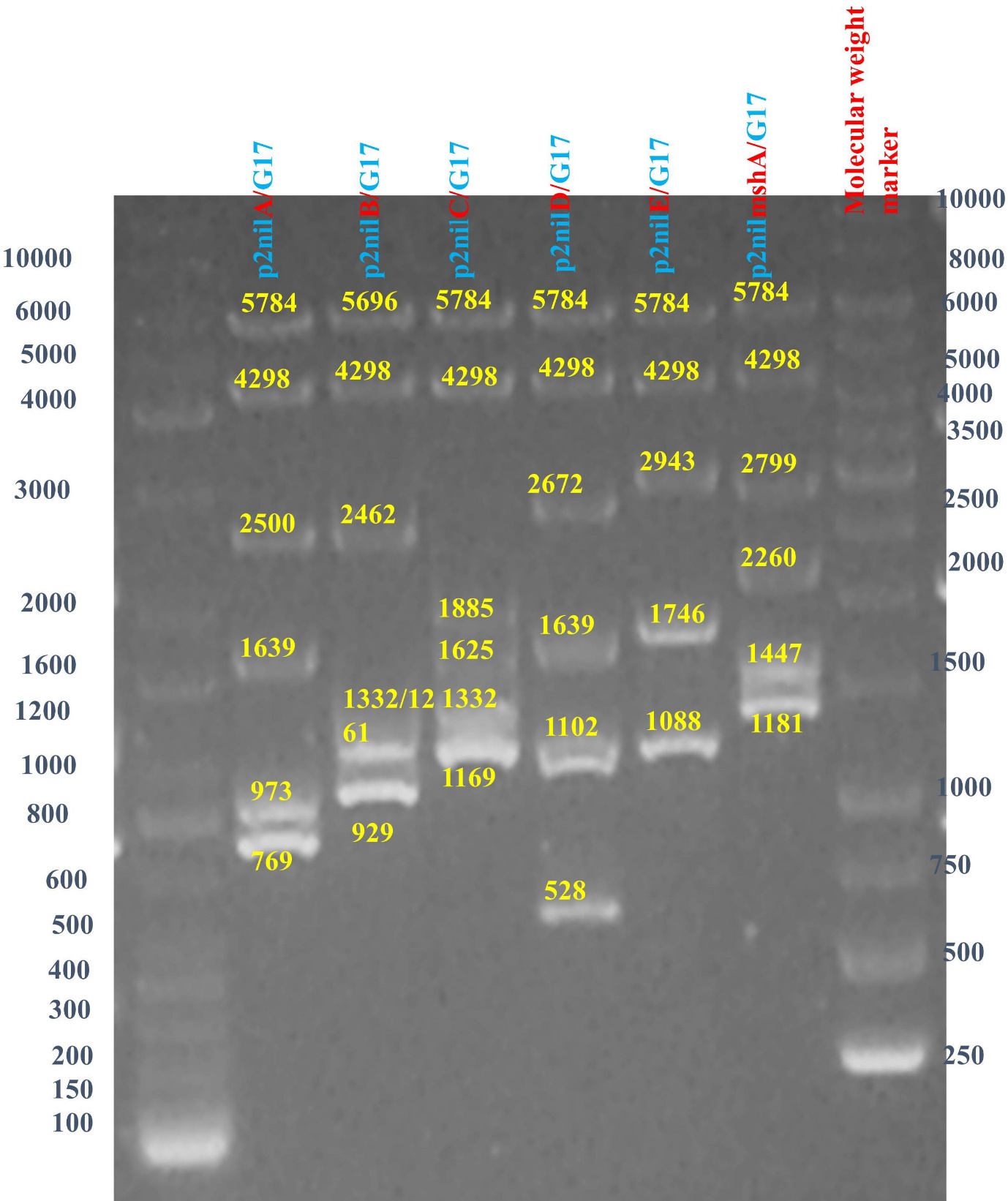


**Figure S9. Restriction enzyme digestion by PstI of the generated** **constructs**. Expected bands according to the map in the program GENTle were obtained (Data Citation 1). Digestion of deletion constructs of the *egtA* second lane, *egtB* third lane, *egtC* fourth lane, *egtD* fifth lane, *egtE* sixth lane and *mshA* seventh lane, and molecular weight markers on the first lane and last name.

# Supplementary Figure. S10


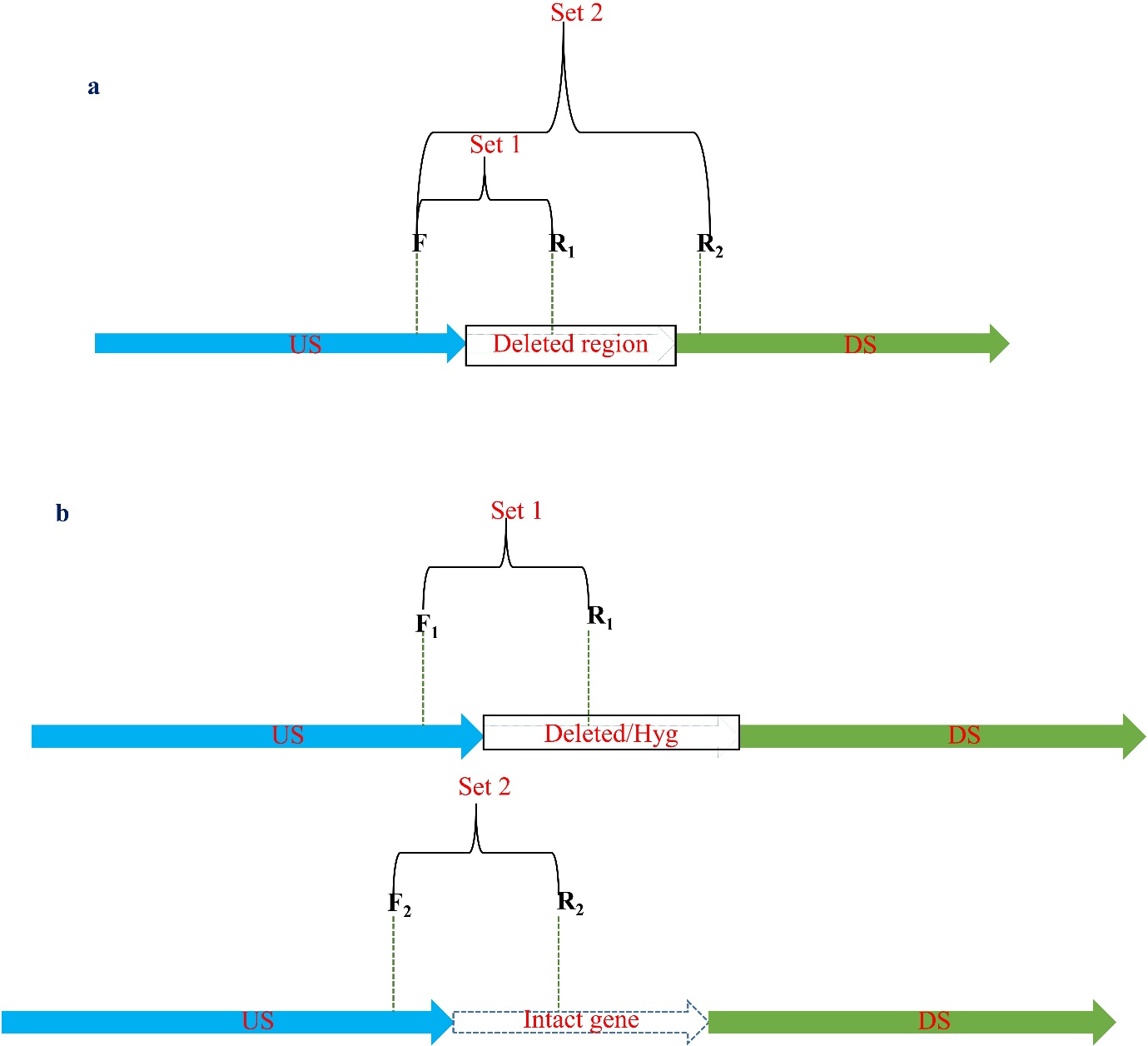


Figure S10. Screening PCR design (a) Screening PCR design of the unmarked mutants, when primer set 1 is used, only the wild type (WT) and single cross over (SCO) are amplified since they both have a copy of the gene, while the mutant is not amplified since the gene has been deleted. When primer set 2 is used, the WT, the SCO and the mutant are amplified, since the reverse primer R_2_ hybridizes outside the deleted region. However, the mutant band is smaller than the wild type band, since the gene has been deleted. (b) Screening PCR design of the marked mutant, when primer set 1 is used, only mutants and SCOs are amplified since they both have the hygromycin (Hyg) cassette, while the WT is not amplified. When primer set 2 is used, the WT and the SCO are amplified but not the mutant, since the reverse primer R_2_ hybridizes in the deleted region.

# Supplementary Figure. S11


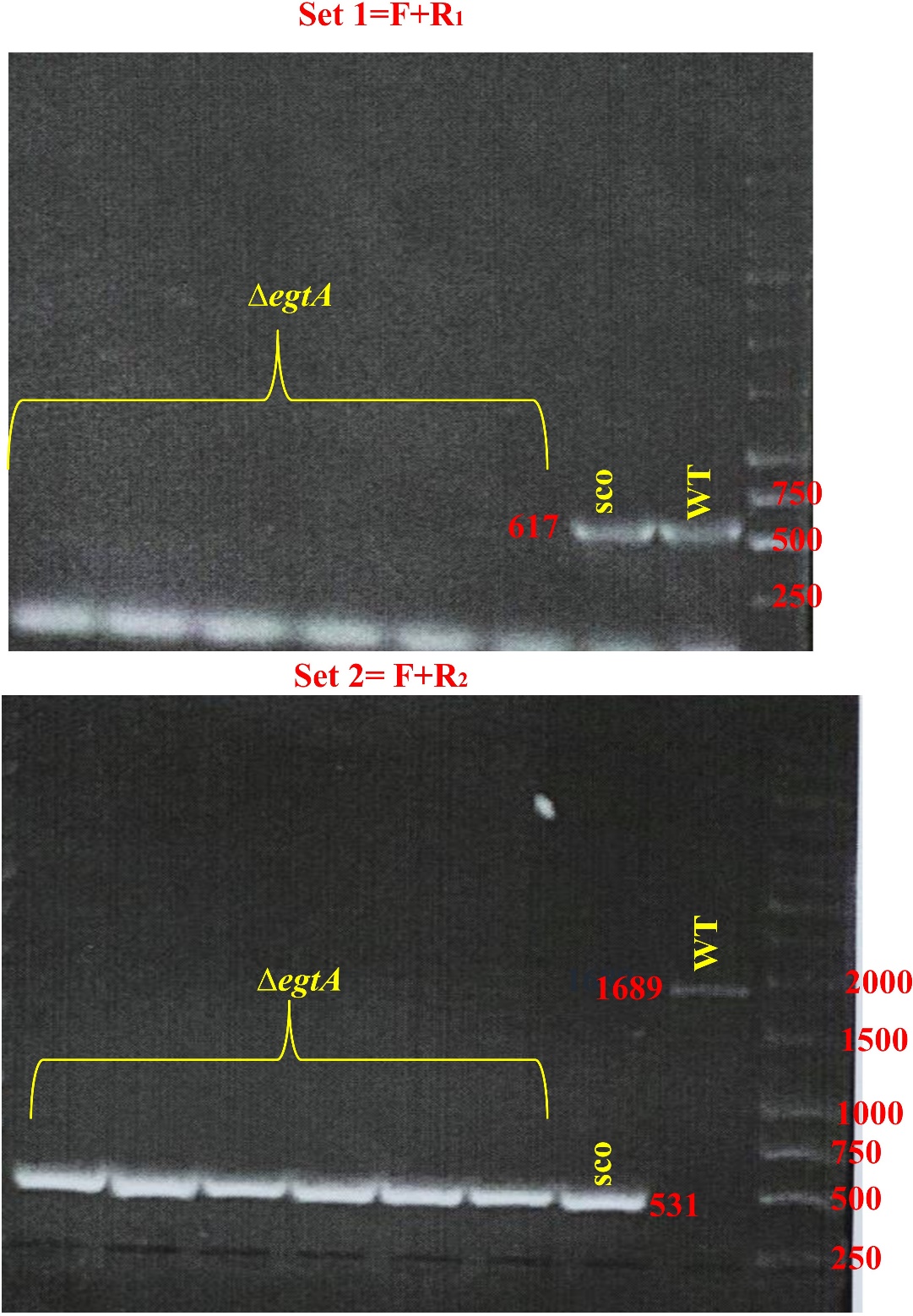


Figure S11. Screening PCR of the Δ*egtA* deletion generated in CDC1551

An 1158bp region of *egtA* was completely deleted from CDC1551, therefore when screened with primer set 1; only primer dimers were observed in the mutants while a 617bp fragment could be amplified in the SCO and WT. When screened with primer set 2, the WT with the intact gene has a 1689bp fragment while the mutant and the SCO have a smaller fragment of 531bp which is 1689bp minus the deleted 1158bp.

# Supplementary Figure. S12


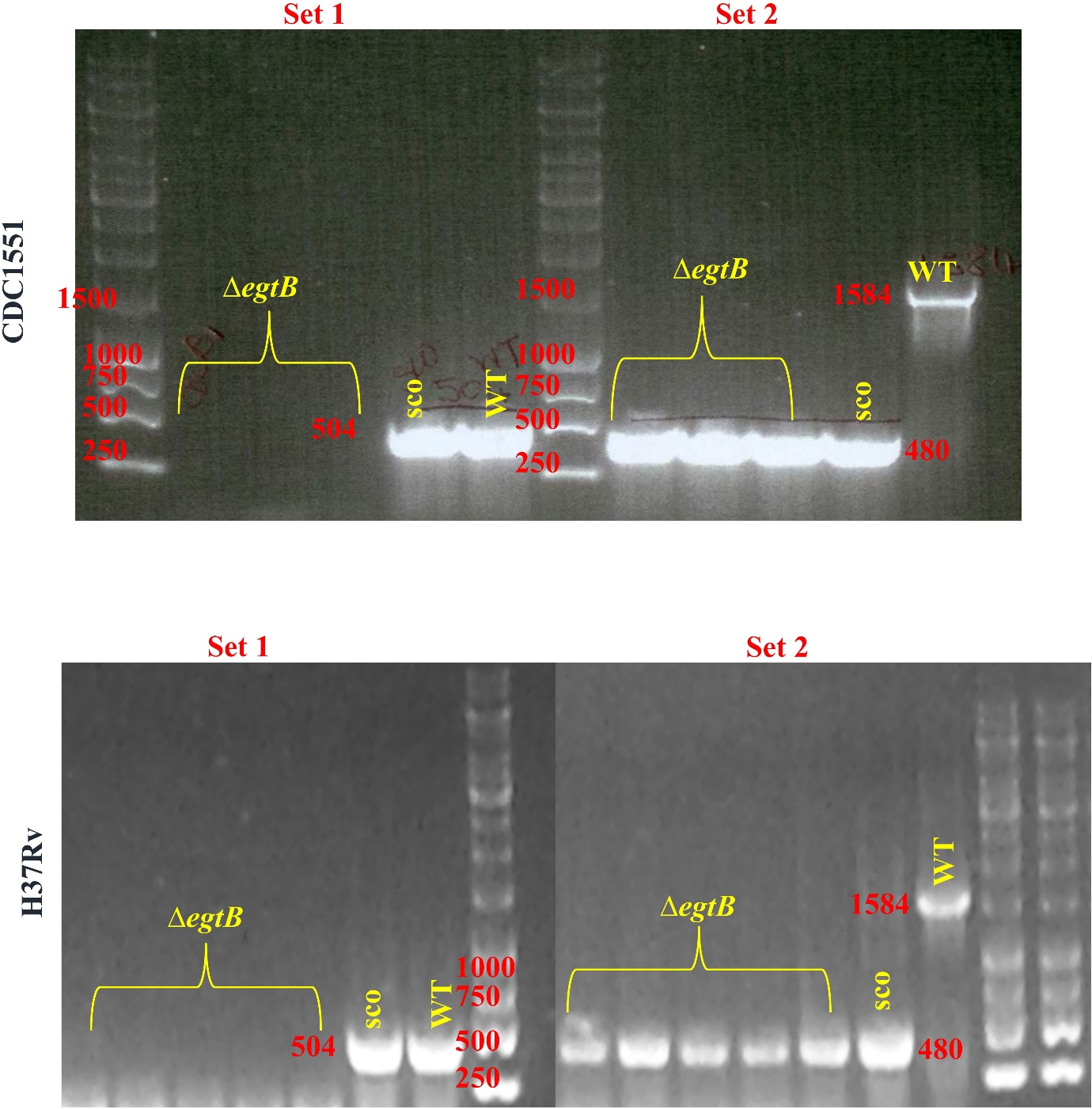


Figure S12. Screening PCR of *egtB* deletion generated in CDC155 and H37Rv. An 1104bp region of *egtB* was completely deleted from CDC1551 and H37Rv, therefore when screened with primer set 1; the mutants could not be amplified while a 504bp fragment could be amplified in the SCO and WT. When screened with primer set 2, the WT with the intact gene has a 1584bp fragment while the mutant and the SCO have a smaller fragment of 480bp which is 1584bp minus the deleted 1104bp.

# Supplementary Figure. S13


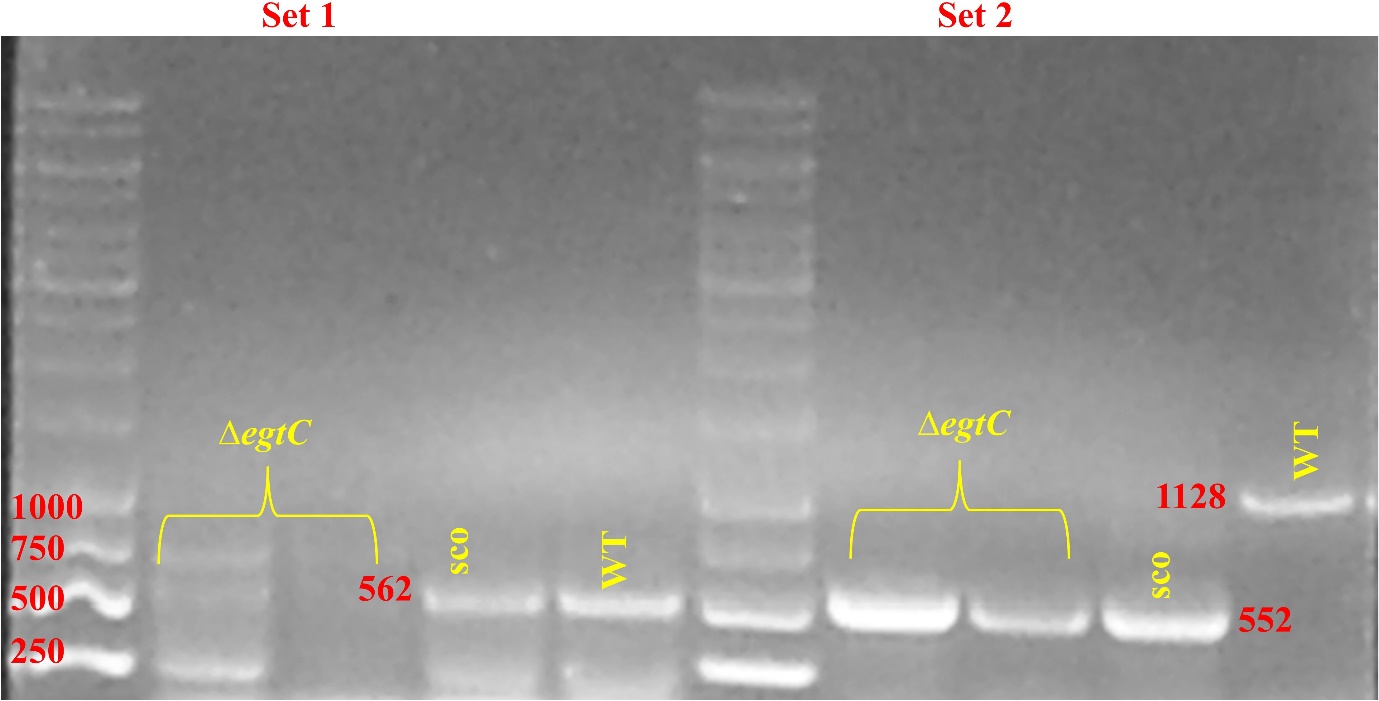


Figure S13. Screening PCR of *egtC* deletion generated in CDC155. A 576bp region of *egtC* was completely deleted from CDC1551, therefore when screened with primer set 1; the mutants could not be amplified while a 562bp fragment could be amplified in the SCO and WT. When screened with primer set 2, the WT with the intact gene has an 1128bp fragment while the mutant and the SCO have a smaller fragment of 552bp which is 1128bp minus the deleted 576bp

# Supplementary Figure. S14


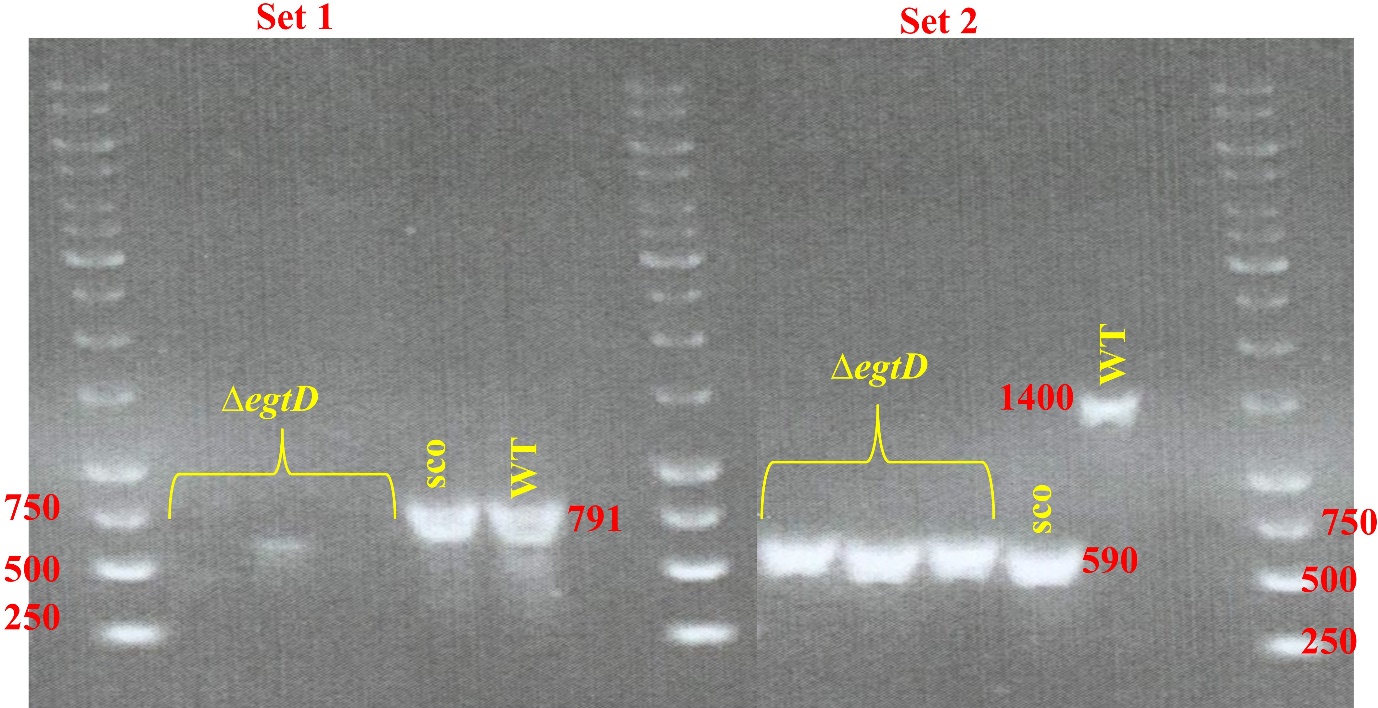


Figure S14. Screening PCR of *egtD* deletion generated in CDC155. An 810bp region of *egtD* was completely deleted from CDC1551, therefore when screened with primer set 1; the mutants could not be amplified while a 791bp fragment could be amplified in the SCO and WT. When screened with primer set 2, the WT with the intact gene has a 1400bp fragment while the mutant and the SCO have a smaller fragment of 590bp which is 1400bp minus the deleted 810bp

# Supplementary Figure. S15


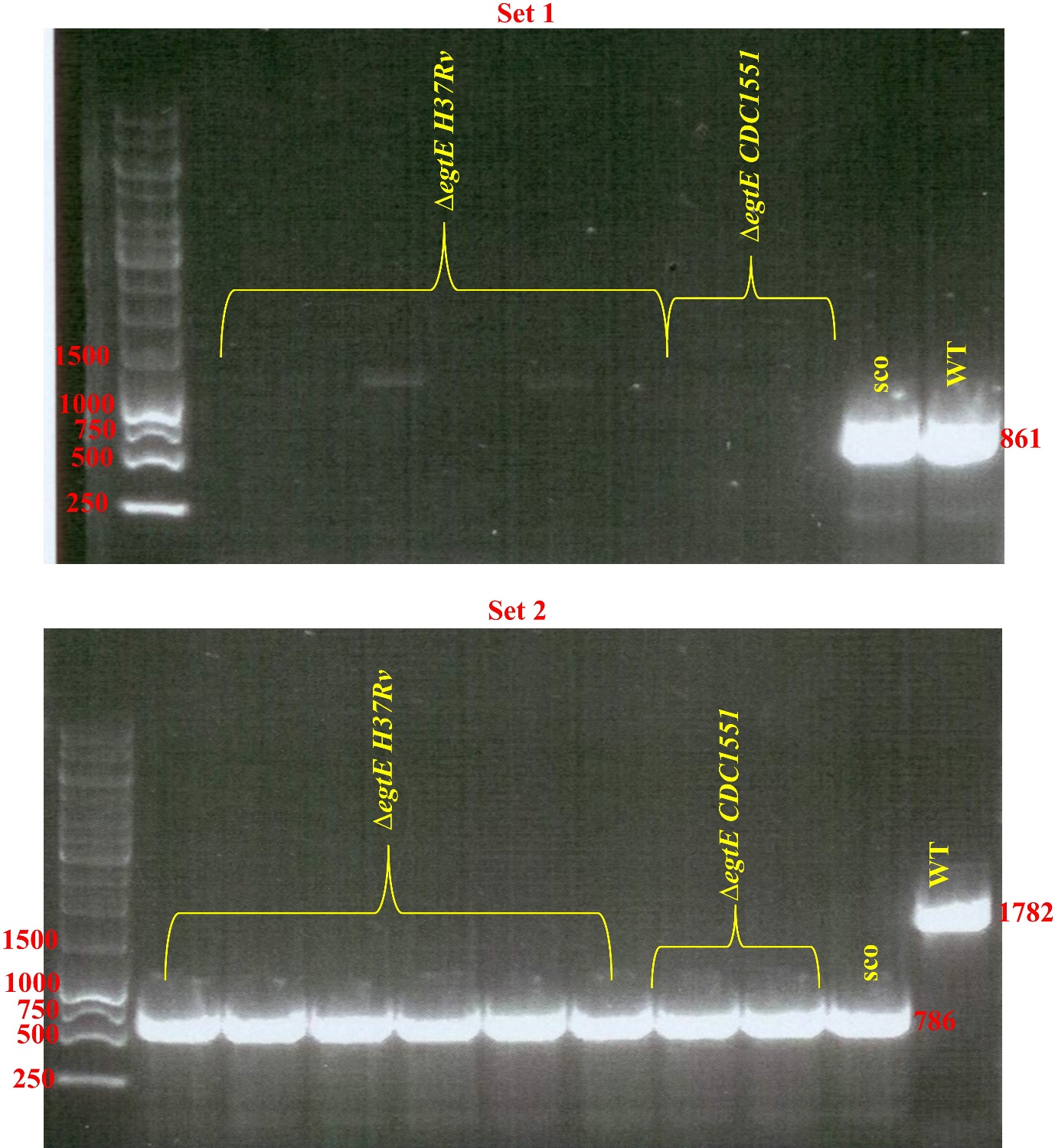


Figure S15. Screening PCR of *egtE* deletion generated in H37Rv and CDC155. A 996bp region of *egtE* was completely deleted from H37Rv and CDC1551, therefore when screened with primer set 1; the mutants could not be amplified while an 861bp fragment could be amplified in the SCO and WT. When screened with primer set 2, the WT with the intact gene has a 1782bp fragment while the mutant and the SCO have a smaller fragment of 786bp which is 1782bp minus the deleted 996bp.

# Supplementary Figure. S16


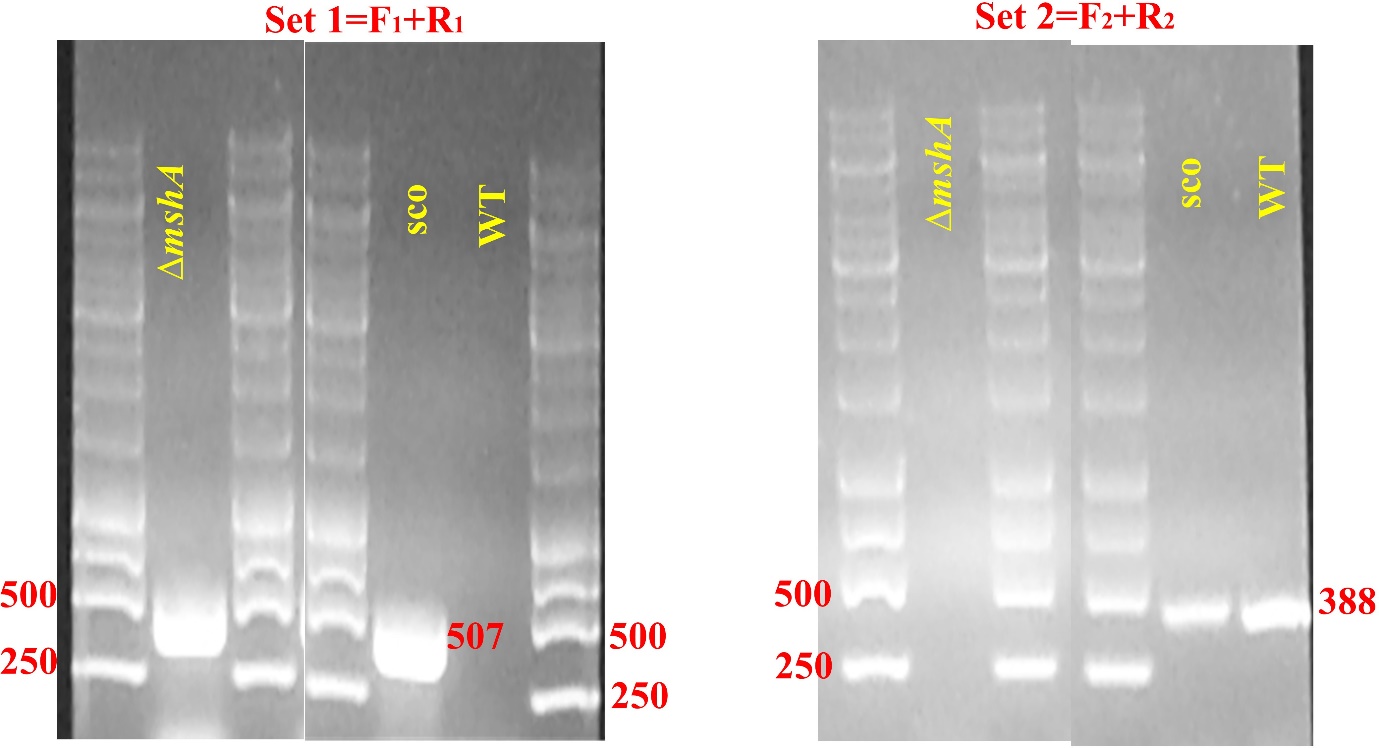


**Figure S16. Screening PCR of *mshA* deletion generated in CDC1551.** A 1335bp region of *mshA* was completely deleted and replace with the hygromycin cassette and its promoter. When primer set 1 that hybridizes outside the deleted region and within the hygromycin cassette is used, only the mutant and the SCO (that still has the construct) are amplified giving a 507bp band. When primer set 2 is used, where R_2_ hybridizes in the intact gene, only the wild type and the SCO are amplified giving a 388bp fragment.

# Supplementary Figure. S17


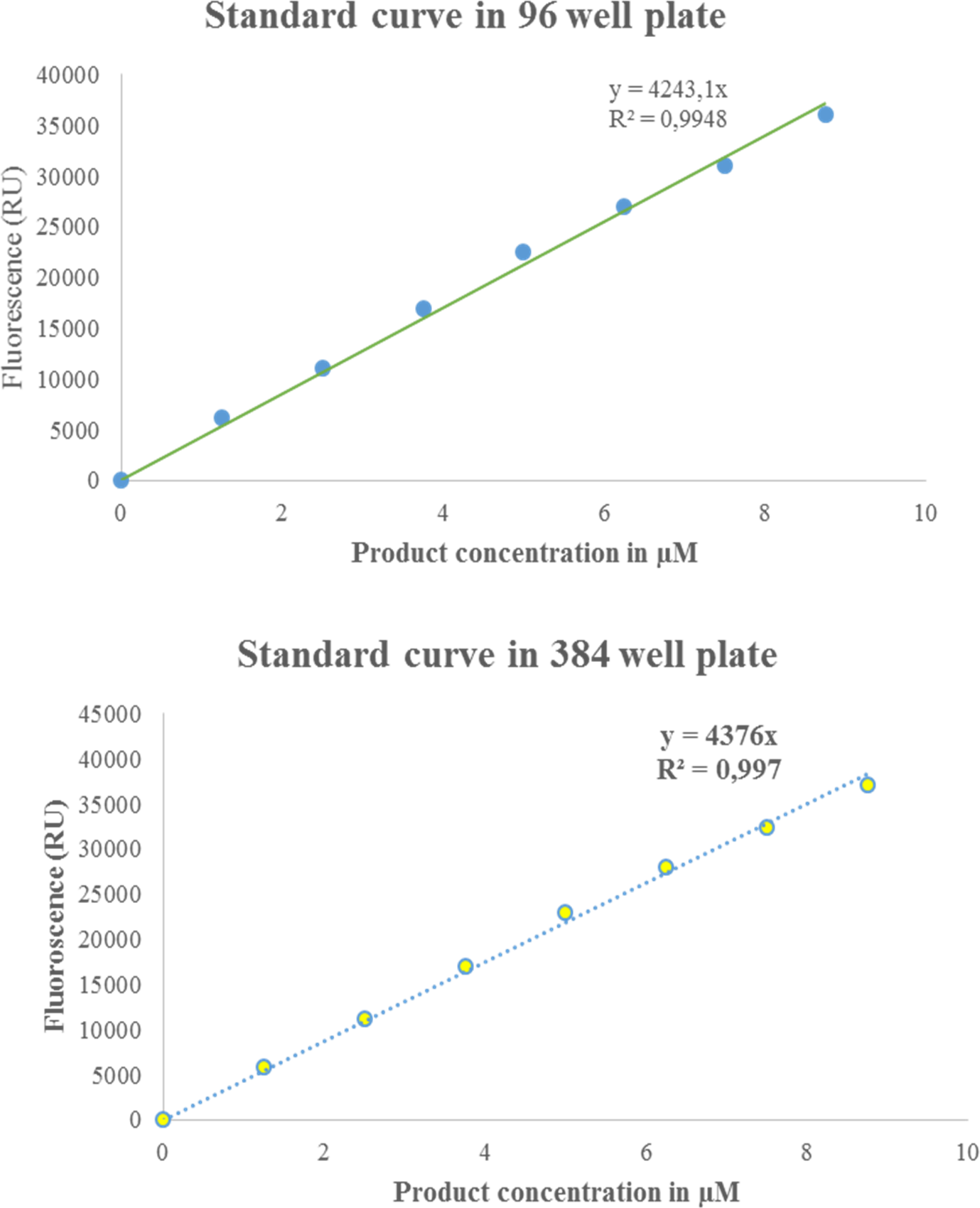


**Figure S17. Investigation of the effect of the reaction volume in the performance of the SAMfluoro™: SAM Methyltransferase Assay kit.** The top panel represents the standard curve obtained from the recommended reaction volume, while the bottom panel represents the standard curve obtained from the volume used in this study (10X less the recommended volume).
